# Mammalian γ2 AMPK regulates intrinsic heart rate

**DOI:** 10.1038/s41467-017-01342-5

**Published:** 2017-11-02

**Authors:** Arash Yavari, Mohamed Bellahcene, Annalisa Bucchi, Syevda Sirenko, Katalin Pinter, Neil Herring, Julia J. Jung, Kirill V. Tarasov, Emily J. Sharpe, Markus Wolfien, Gabor Czibik, Violetta Steeples, Sahar Ghaffari, Chinh Nguyen, Alexander Stockenhuber, Joshua R. St. Clair, Christian Rimmbach, Yosuke Okamoto, Dongmei Yang, Mingyi Wang, Bruce D. Ziman, Jack M. Moen, Daniel R. Riordon, Christopher Ramirez, Manuel Paina, Joonho Lee, Jing Zhang, Ismayil Ahmet, Michael G. Matt, Yelena S. Tarasova, Dilair Baban, Natasha Sahgal, Helen Lockstone, Rathi Puliyadi, Joseph de Bono, Owen M. Siggs, John Gomes, Hannah Muskett, Mahon L. Maguire, Youlia Beglov, Matthew Kelly, Pedro P. N. dos Santos, Nicola J. Bright, Angela Woods, Katja Gehmlich, Henrik Isackson, Gillian Douglas, David J. P. Ferguson, Jürgen E. Schneider, Andrew Tinker, Olaf Wolkenhauer, Keith M. Channon, Richard J. Cornall, Eduardo B. Sternick, David J. Paterson, Charles S. Redwood, David Carling, Catherine Proenza, Robert David, Mirko Baruscotti, Dario DiFrancesco, Edward G. Lakatta, Hugh Watkins, Houman Ashrafian

**Affiliations:** 1 Experimental Therapeutics, Radcliffe Department of Medicine, University of Oxford, Oxford, OX3 9DU UK; 2 Division of Cardiovascular Medicine, Radcliffe Department of Medicine, University of Oxford, Oxford, OX3 9DU UK; 3 The Wellcome Trust Centre for Human Genetics, Oxford, OX3 7BN UK; 4 Department of Biosciences, Università degli Studi di Milano, Milan, 20133 Italy; 5 Centro Interuniversitario di Medicina Molecolare e Biofisica Applicata, University of Milano, Milan, 20133 Italy; 6 Laboratory of Cardiovascular Science, Intramural Research Program, National Institute on Aging, NIH, Baltimore, MD 21224 USA; 7 Burdon Sanderson Cardiac Science Centre, Department of Physiology, Anatomy & Genetics, University of Oxford, Oxford, OX1 3PT UK; 8 Department of Cardiac Surgery, Rostock University Medical Centre, 18057 Rostock, Germany; 9 Department Life, Light and Matter, Interdisciplinary Faculty, Rostock University, 18059 Rostock, Germany; 10 Department of Physiology and Biophysics, University of Colorado School of Medicine, Aurora, CO 80045 USA; 11 Department of Systems Biology and Bioinformatics, University of Rostock, Rostock, 18051 Germany; 12 MRC Human Immunology Unit, Weatherall Institute for Molecular Medicine, Nuffield Department of Medicine, University of Oxford, Oxford, OX3 9DS UK; 13 Department of Medicine, BHF Laboratories, The Rayne Institute, University College London, London, WC1E 6JJ UK; 14 Instituto de Pós-Graduação, Faculdade de Ciências Médicas de Minas Gerais, Belo Horizonte, 30.130-110 Brazil; 15 Cellular Stress Group, MRC London Institute of Medical Sciences, Imperial College London, London, W12 0NN UK; 16 Nuffield Department of Clinical Laboratory Science, University of Oxford, Oxford, OX3 9DU UK; 17 The Heart Centre, William Harvey Research Institute, Barts and the London School of Medicine and Dentistry, London, EC1M 6BQ UK; 18 Stellenbosch Institute of Advanced Study (STIAS), Wallenberg Research Centre at Stellenbosch University, Stellenbosch, 7602 South Africa

## Abstract

AMPK is a conserved serine/threonine kinase whose activity maintains cellular energy homeostasis. Eukaryotic AMPK exists as αβγ complexes, whose regulatory γ subunit confers energy sensor function by binding adenine nucleotides. Humans bearing activating mutations in the γ2 subunit exhibit a phenotype including unexplained slowing of heart rate (bradycardia). Here, we show that γ2 AMPK activation downregulates fundamental sinoatrial cell pacemaker mechanisms to lower heart rate, including sarcolemmal hyperpolarization-activated current (*I*_f_) and ryanodine receptor-derived diastolic local subsarcolemmal Ca^2+^ release. In contrast, loss of γ2 AMPK induces a reciprocal phenotype of increased heart rate, and prevents the adaptive intrinsic bradycardia of endurance training. Our results reveal that in mammals, for which heart rate is a key determinant of cardiac energy demand, AMPK functions in an organ-specific manner to maintain cardiac energy homeostasis and determines cardiac physiological adaptation to exercise by modulating intrinsic sinoatrial cell behavior.

## Abstract (web summary)

AMPK regulates cellular energy balance using its γ subunit as an energy sensor of cellular AMP and ADP to ATP ratios. Here, the authors show that γ2 AMPK activation lowers heart rate by reducing the activity of pacemaker cells, whereas loss of γ2 AMPK increases heart rate and prevents the adaptive bradycardia of endurance training in mice.

## Introduction

Cellular survival and function depend fundamentally on the liberation of free energy, achieved through the intermediary process of ATP hydrolysis, to drive anabolic processes. Cells have to contend with the challenge of fluctuating internal bioenergetic demands and environmental substrate availability, necessitating a precise system of monitoring cellular energetic status dynamically. In eukaryotic cells, the adenylate charge-regulated sensor/effector arm of this system is recognized to be AMP-activated protein kinase (AMPK), a serine/threonine kinase existing as a heterotrimeric complex of catalytic α and regulatory β and γ subunits^1^. The γ AMPK subunit—existing in mammals as three isoforms (γ1, γ2, and γ3)—confers energy sensing on the holoenzyme through competitive binding of adenyl nucleotides at the interfaces of four tandem cystathionine β-synthase (CBS) repeats^2^. AMPK is largely inactive under physiological conditions due to Mg.ATP complexing^2^; however, in response to energetic stress — corresponding to increased AMP/ATP and/or ADP/ATP — AMPK activates catabolic ATP–generating metabolic processes and represses anabolic functions via direct phosphorylation and altered transcription^3^, restoring cellular energy charge.

In contrast to unicellular eukaryotes, metazoans must ensure energy homeostasis for the organism as a whole, coupling energy (i.e., food) intake to metabolic requirements of diverse multi-cellular tissues^4^. This transition necessitated the evolutionary co-option of AMPK from a primordial cell-autonomous fuel gauge to a systemic energy regulator responsive to multiple hormonal and nutritional cues^5^, exemplified by the contribution of hypothalamic AMPK to the central effect of hormones influencing feeding behavior, such as ghrelin^6^, leptin^7^, and thyroid hormone^8^. In both mice and humans, activating mutations in γ2 AMPK that alter hypothalamic orexigenic neuronal excitability and drive caloric surfeit^9^ suggest that AMPK activity, influenced by the nucleotide sensing γ subunit, has adapted in these highly specialized cells to function as a systemic energy sensor, defending the metabolic requirements of the entire organism. However, a role for regulation of organ-specific caloric accounting by AMPK activity in cell populations beyond such central neuronal circuits has not been reported.

The heart exhibits the highest organ-specific resting metabolic rate of any organ^10^ and remarkable energetic stewardship, with the highest work increment of any organ, achieving dynamic workload of 5000–50,000 mmHg beats/min, typically at very high heart rates (HRs), without any increase in free ADP levels. HR scales linearly with myocardial oxygen consumption, with the latter further increased by the enhanced contractility accompanying an increased HR^11^. As the background HR is set by the intrinsic automaticity of the cardiac pacemaker—a population of highly specialized sinoatrial (SA) cells—these observations suggest a direct link between SA cell firing rate and cardiac energy homeostasis.

In humans, activating mutations in the gene encoding the γ2 subunit of AMPK (*PRKAG2*) result in an autosomal dominant disorder whose heterogeneous phenotypic spectrum includes left ventricular hypertrophy (LVH) and prominent electrophysiological disturbances^12,13^. Cardiac-restricted transgenic mouse models overexpressing human *PRKAG2* mutations recapitulate major aspects of this spectrum, including severe LVH, ventricular pre-excitation, and propensity to sudden death^14,15^. Histological analyses of hearts from these models and human mutation carriers have identified cardiomyocyte glycogen accumulation and suggested a direct role for glycogen in the pathogenesis of pre-excitation^14^. However, the prominent sinus bradycardia, which contributes to the substantial requirement for early pacemaker implantation, remains poorly understood^13,16,17^.

Here, we use a combination of genetic, electrophysiological, transcriptomic, and cellular approaches applied to genetic models of altered AMPK function to examine its role in the regulation of the mammalian cardiac pacemaker. Our results reveal an important organ-specific function for γ2 AMPK in the regulation of intrinsic SA cell firing rate in health and disease, linking this conserved cellular energy sensor to the control of mammalian SA node and thereby myocardial energy homeostasis through its influence on HR.

## Results

### γ2 AMPK activation induces sinus bradycardia

The majority of cardiomyopathy-causing *PRKAG2* mutations are missense substitutions of highly conserved residues within or in close proximity to the CBS motifs of the γ2 subunit of AMPK^18^. Studies of transgenic mice and acute viral transduction experiments^19^ suggest that the primary effect of these γ2 mutations to be basal activation of the enzyme complex, likely due to a failure to adequately sense inhibitory ATP. Furthermore, the relative proportions of the different γ subunits appear to be important; for example, even overexpression of wild-type (WT) γ2 has been associated with a cardiac phenotype in mice, including LVH and glycogen excess^14^. This may reflect altered γ isoform stoichiometry (i.e., the γ1/γ2 ratio, with γ1 representing the physiologically predominant cardiac isoform)^20^.

**Fig. 1:**
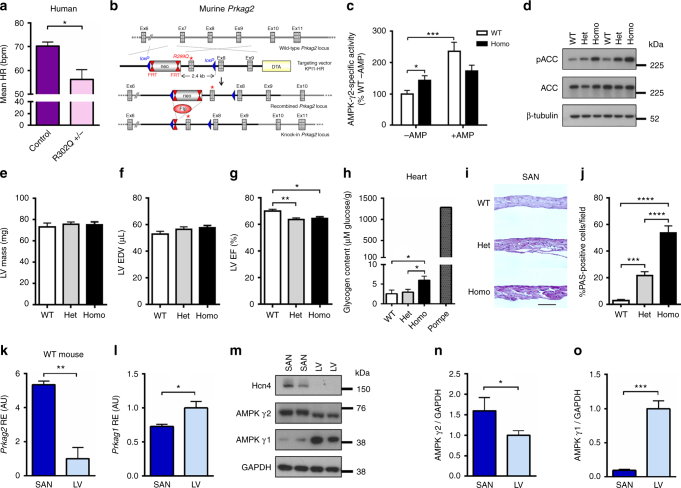
Generation of the R299Q γ2 AMPK knock-in mouse and enrichment of γ2 AMPK in WT SA nodes. **a** Mean 24-h heart rate (HR) of human heterozygous R302Q γ2 mutation carriers (age 41.2 ± 2.8 years) vs genotype-negative sibling controls (age 38.9 ± 2.3 years) (*n* = 10–15). All subjects had any anti-arrhythmic drugs or β-adrenoceptor blockers discontinued for 5 days prior to ECG and none were on amiodarone. **b** Schematic of gene-targeting strategy to generate the R299Q γ2 AMPK knock-in. Neo, neomycin selection cassette; FRT, Flp recombinase recognition target; red asterisk denotes mutation in exon 7 of *Prkag2*. **c** γ2 AMPK-specific activity of freeze-clamped ex vivo perfused hearts measured by SAMS peptide phophorylation assay in the absence or presence of AMP (*n* = 18–22). **d** Representative western blot of whole heart tissue from R299Q γ2 and WT mice for phospho-(p) ACC (*n* = 11–15). **e**–**g** Cine MRI analysis of left ventricular (LV) mass (**e**), end-diastolic volume (EDV) (**f**) and ejection fraction (EF) (**g**) in R299Q γ2 and WT mice aged 2 months (*n* = 8–19). **h** Cardiac tissue glycogen content from 12 month R299Q γ2 and WT mice together with a positive control heart from a homozygous *Gaa* (encoding acid α-glucosidase) knockout mouse (*n* = 12–15). **i**, **j** Periodic acid-Schiff (PAS) staining (**i**) (scale bar, 5 µm) and quantification of glycogen content (**j**) (as %PAS-positive cells/field) of SA node (SAN) sections (*n* = 12). **k**, **l** Quantitative real-time PCR (qRT-PCR) of γ2 and γ1 AMPK isoform relative gene expression levels (normalized to β-actin) from normal murine SA node and LV (SAN, *n* = 4; LV, *n* = 10). **m**–**o** Western blot (**m**) and densitometry (**n**, **o**) of γ2 and γ1 AMPK in normal murine SA node and LV, together with SA node positive (HCN4) and loading (GAPDH) controls (*n* = 6–8). Uncropped western blots are shown in Supplementary Fig. 10. **a**, **k**, **l**, **n**, **o** Student’s *t*-test was performed. **c** Kruskal–Wallis test followed by Dunn’s multiple comparisons test was performed. **e**–**h**, **j** One-way analysis of variance (ANOVA) followed by Holm–Sidak’s multiple comparisons test was performed. *P < 0.05, **P < 0.01, ***P < 0.001, ****P < 0.0001. RE relative expression, AU arbitrary units. **a**, **c**, **e**–**h**, **j**–**l**, **n**, **o** Data are shown as means ± s.e.m.

We observed that humans carrying the R302Q mutation in *PRKAG2* (the most frequently described) exhibit sinus bradycardia with a significantly lower resting HR compared with genotype-negative sibling controls (Fig. 1a). To gain insights into the pathogenesis of *PRKAG2*-related sinus bradycardia free of confounders inherent to overexpression transgenesis, we used gene-targeted mice with the R299Q mutation (orthologous to R302Q in humans) introduced into murine *Prkag2*, permitting the expression and regulation of mutant protein under endogenous control mechanisms (Fig. 1b)^9^. Mice heterozygous (Het) for the R299Q γ2 mutation were interbred to generate WT and homozygous (Homo) mice. Competitive multiplex PCR confirmed expression of R299Q γ2 transcripts in mutant but not WT mice (Supplementary Fig. 1a). Western blotting confirmed comparable cardiac γ2 and γ1 expression across genotypes (Supplementary Fig. 1b–d). Cardiac γ2-specific basal AMPK activity was increased in R299Q γ2 mice compared with WT (Fig. 1c). Consistent with its proximity to the nucleotide-binding site^2^ and previous reports^18,21^, activation of R299Q γ2 AMPK complexes by AMP was limited compared with WT (Fig. 1c). In R299Q γ2 hearts, a corresponding increase in the phosphorylation of acetyl-CoA carboxylase at Ser79, a canonical AMPK target, was observed (Fig. 1d; Supplementary Fig. 1e), consistent with a basal gain-of-function of γ2 AMPK complexes in vivo.

Cine MRI revealed no evidence for LVH or LV dilatation, but R299Q γ2 mice exhibited a subtle reduction in contractile performance at 2 months of age (Fig. 1e–g), with no progression at 10 months (Supplementary Fig. 1f–i). Cardiac energetics, as determined by in vivo ^31^P-MRS measurement of the phosphocreatine/γ-ATP ratio, was unaltered at 2 months (Supplementary Fig. 1j). Cardiac histology and ultrastructure of R299Q γ2 mice appeared indistinguishable from WT mice (Supplementary Fig. 2a, c). We found a small increase in biochemical cardiac glycogen content in homozygous R299Q γ2 mice at 12 months (Fig. 1h), associated with upregulation of several genes involved in glucose transport (*Slc2a1*, *Slc2a4*) and glycogen metabolism (*Gyg*, *Pygm*) (Supplementary Fig. 2b).

In contrast to findings in whole heart, detailed regional histological analysis of SA node sections revealed a striking excess of glycogen in R299Q γ2 mice (Fig. 1i, j; Supplementary Fig. 2h–j), with increased maximal SA node thickness (Supplementary Fig. 2e) but otherwise unremarkable histological appearances (Supplementary Fig. 2d, f, g), including no evidence of apoptosis on TUNEL staining, suggesting correspondingly greater AMPK activation^22^ in the SA node. Accordingly, we assessed γ isoform transcript expression in normal murine SA nodes compared to left ventricles (LVs) and found *Prkag2*, but not *Prkag1*, to be enriched in SA nodes (Fig. 1k, l). We observed corresponding SA node enrichment of γ2 protein, but a striking paucity of γ1, suggesting that γ2 is the predominant γ isoform in this tissue (Fig. 1m–o; Supplementary Fig. 1k–m). We also observed significantly lower expression of α2 AMPK in the SA node compared to the LV (Supplementary Fig. 1n, o).

**Fig. 2:**
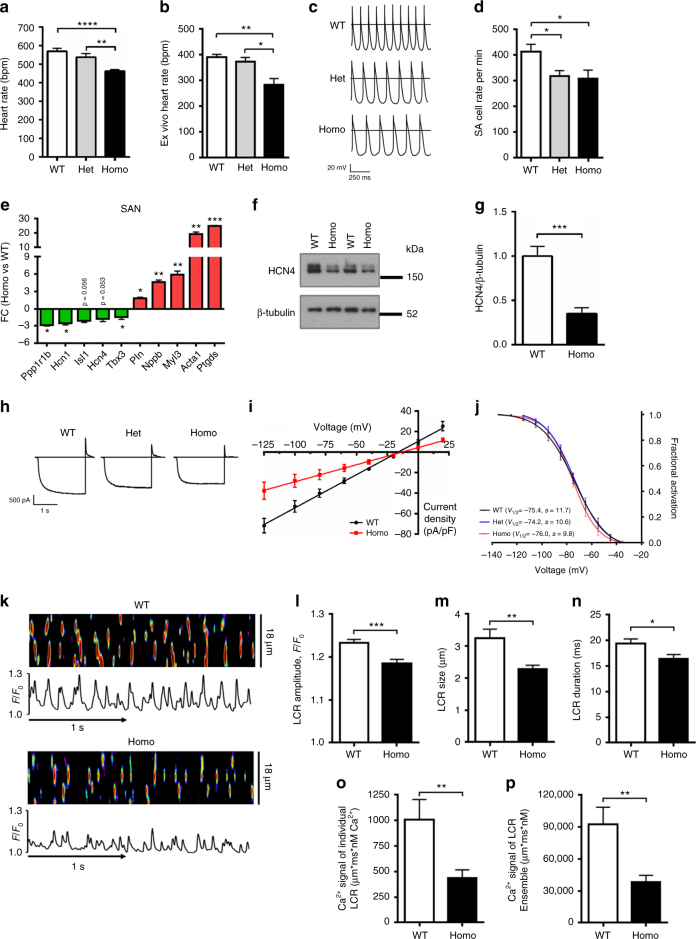
γ2 AMPK activation lowers intrinsic HR by downregulating SA cell *I*_f_ and Ca^2+^ clock pacemaker mechanisms. **a** HR in beats per minute (bpm) of R299Q γ2 and WT mice under anesthesia (*n* = 7–12). **b** HR during ex vivo-isolated cardiac perfusion (*n* = 6–11). **c** Representative action potentials from SA cells isolated from R299Q γ2 and WT mice. **d** Mean beating rate of SA cells from groups illustrated in **c** (*n* = 17 cells). **e** qRT-PCR validation of differentially expressed genes on SA node microarray (*n* = 3). FC fold-change. **f**, **g** Representative western blot (**f**) and analysis (**g**) of HCN4 levels in SA nodes from R299Q γ2 and WT mice. **h** Representative SA cell *I*_f_ traces during steps to −125 mV. **i** Mean fully activated *I*/*V* curves (*I*_f_ density plotted against membrane voltage) recorded in WT and R299Q γ2 SA cells. Linear data fitting yielded significant differences (*P* < 0.0001) in *I*_f_ slope conductance (648 and 333 pS/pF for WT and homozygous R299Q γ2 SA cells, respectively) (*n* = 8–10 cells/2–6 mice). **j** Mean voltage dependence of *I*_f_ activation of WT and R299Q γ2 SA cells (*n* = 6 per genotype). Half-activation voltages (*V*_1/2_, mV) and inverse-slope factors (*s*, mV) depicted. **k** Representative confocal line-scan images and Ca^2+^ transients of isolated, single, permeabilized WT, and homozygous R299Q γ2 SA node cells bathed in 50 nmol/L free [Ca^2+^]. **l**–**n** Mean spontaneous local Ca^2+^ release (LCR) amplitude (**l**) expressed as peak value (*F*) normalized to minimal (*F*_0_) fluorescence, size (**m**), and duration (**n**). **o**, **p** Ca^2+^ signal of individual LCR (**o**) and LCR ensembl (**p**) (*n* = 15–17 cells/3 mice per genotype). Uncropped western blots are shown in Supplementary Fig. 10. **a**, **b**, **d** One-way ANOVA followed by Holm–Sidak’s multiple comparisons test was performed. **i** Comparison of the slopes of linear regression lines was performed. **e**, **g**, **l**–**p** Student’s *t*-test was performed. *P < 0.05, **P < 0.01, ***P < 0.001, ****P < 0.0001. **a**, **b**, **d**, **e**, **g**, **l**–**p** Data are shown as means ± s.e.m.

Reminiscent of the sinus bradycardia of human R302Q *PRKAG2* mutation carriers (Fig. 1a), invasive electrophysiology studies performed under isoflurane general anesthesia revealed a reduction in sinus HR of homozygous R299Q γ2 mice in vivo (Fig. 2a). The PR interval and anterograde atrioventricular conduction parameters were not significantly different to WT, with no evidence of ventricular pre-excitation either at baseline or with programmed atrial stimulation (Supplementary Table 1). Ambulatory HR recordings confirmed invasive findings, with marked sinus bradycardia in homozygous R299Q γ2 compared with WT mice (416 ± 13 bpm vs 549 ± 15 bpm, respectively, *P* < 0.0001, Student’s *t*-test; Supplementary Fig. 3a, b).

### γ2 AMPK activation reduces SA cell automaticity

Assessment of isolated perfused hearts from R299Q γ2 mice demonstrated a lower intrinsic HR (Fig. 2b). As a corollary, isolated SA cells from R299Q γ2 mice exhibited reduced basal firing rate but unaltered maximum diastolic potential (MDP) (Fig. 2c, d; Supplementary Fig. 3c). We next measured SA cell rate responses to catecholamine and muscarinic stimulation. We observed significant increases in SA cell firing rates in response to the β-adrenergic receptor agonist isoproterenol in homozygous R299Q γ2 mice, with a magnitude of change from baseline comparable to WT mice, thereby reaching a marginally lower maximal rate (Supplementary Fig. 3e). Both genotypes exhibited profound reductions in SA cell firing rates in response to the endogenous muscarinic receptor agonist acetylcholine (ACh) (Supplementary Fig. 3f). These data indicate that the activating R299Q γ2 AMPK mutation, in the context of a broadly healthy SA node with chronotropic competence, induces intrinsic sinus bradycardia in mice by reducing the basal SA cell firing rate while retaining high responsivity to catecholamine and cholinergic rate modulation.

### γ2 AMPK activation reprograms the SA node transcriptome

To delineate the molecular mechanisms contributing to the intrinsic sinus bradycardia of R299Q γ2 mice, we obtained SA node gene expression profiles and identified significant differences in gene expression (Supplementary Fig. 4; Supplementary Tables 2 and 3). These included: changes suggestive of a transition of the SA node to a less nodal phenotype (upregulation in *Myl2*, *Myl3*, *Nppb*, and *Tnnt3*); downregulation of transcriptional regulators critical to SA node development and function (*Isl1* and *Tbx3*)^23,24^; alterations in constituents of the sarcolemmal membrane voltage clock (upregulation in *Kcna5* and *Kcnv2*); genes involved in Ca^2+^ homeostasis or regulation of the sarcoplasmic reticulum (SR) Ca^2+^ clock (upregulation in *Parv*, *Pln*, and downregulation in *Atp2a1*, *Calr*, *Casq1*, and *Ppp1r1b*); and genes related to AMPK’s canonical function (i.e., altered expression of genes involved in glucose homeostasis and glycogen metabolism with upregulation in *Fbp2*, *Ganc*, *Pfkfb2*, *Phkb*, *Pgm3*, and downregulation in *Pygm* and *Hk1*). These genes were observed to cluster around and interact with (Supplementary Figs. 5–7) a network of key regulators of pacemaker clock function, including genes encoding cAMP-activated protein kinase (*Pka*) (Supplementary Fig. 5, network 2; Supplementary Fig. 6, network 5), short stature homeobox 2 (*Shox2*) (Supplementary Fig. 6, network 2), the cardiac ryanodine receptor (*Ryr2*), and the calcium and calmodulin-dependent protein kinase II (*CamkII*) (Supplementary Fig. 6, network 6). Quantitative real-time PCR (qRT-PCR) of SA nodes confirmed many of these changes (Fig. 2e), suggesting that γ2 AMPK activation has a transcriptional influence to remodel the coupled-clock and accounting for the observed changes in SA node function.

### γ2 AMPK activation downregulates SA *I*_f_ and spontaneous LCRs

The transcription factors ISL1 and TBX3 critically promote the SA cell-specific gene program^23,24^, including expression of *Hcn4*. HCN4, highly expressed in the mammalian SA node, is one of four hyperpolarization-activated cyclic nucleotide-gated channel isoforms constituting f-channels which are responsible for the sarcolemmal hyperpolarization-activated “funny” current, *I*_f_, critically contributing to the spontaneous depolarization of pacemaker cells, and whose reduced expression is associated with sinus bradycardia^25^. We measured SA node HCN4 protein expression and found a marked reduction in homozygous R299Q γ2 mice (Fig. 2f, g; Supplementary Fig. 3d). Patch-clamping of isolated homozygous R299Q γ2 SA cells revealed a reduction in *I*_f_ density with a substantial and significant reduction in whole-cell *I*_f_ conductance compared with WT (Fig. 2h, i), but no alteration in the *I*_f_ voltage-dependence of activation (Fig. 2j), supporting the contribution of lower f-channel density to reduced SA node *I*_f_. SA cells from both genotypes exhibited similar shifts in the *I*_f_ activation curve upon β-adrenoceptor or muscarinic stimulation using isoproterenol (Iso) or acetylcholine (ACh), respectively, suggesting unperturbed *I*_f_ modulation by these agonists (Supplementary Fig. 3g, h).

Spontaneous rhythmic SR local Ca^2+^ release (LCR) also crucially influences SA cell automaticity^26^. Given the gene expression profile findings, we undertook confocal imaging of LCRs in individual permeabilized WT and R299Q γ2 SA cells (in which the impact of HCN4 and other sarcolemmal electrogenic molecules constituting the membrane clock are uncoupled from the Ca^2+^ clock) bathed in fixed physiologic free [Ca^2+^]. We found significantly lower mean LCR amplitude, size, and duration in R299Q γ2 vs WT SA cells (Fig. 2k–n), resulting in a >50% lower spontaneous Ca^2+^ signal of individual and ensemble LCRs (Fig. 2o, p), which activate the Na^+^/Ca^2+^ exchanger current (*I*_ncx_) in intact SA cells during diastolic depolarization. Consistent with the transcriptome data, immunohistochemistry of isolated SA cells from homozygous R299Q γ2 mice revealed signals for greater phospholamban (PLN) protein expression (Supplementary Fig. 3i), a key negative modulator of LCR periodicity via its inhibitory effects on the SR Ca^2+^ uptake pump, sarco(endo)plasmic reticulum Ca^2+^-ATPase (SERCA)^26^. We verified this finding quantitatively using western blotting, confirming increased phospholamban content in homozygous R299Q γ2 SA nodes compared with WT SA nodes, both in absolute terms and when expressed relative to its cognate protein, SERCA (Supplementary Fig. 3j, l), consistent with reduced SR Ca^2+^ replenishment. However, we identified no significant effect of the R299Q γ2 mutation on levels of other key constituents of spontaneous intracellular Ca^2+^ cycling contributing to pacemaker function, including SERCA itself, calsequestrin (CASQ1), the cardiac ryanodine receptor (RYR2), the Na^+^/Ca^2+^exchanger (NCX1), or L-type Ca^2+^ channels (LTCC) (Supplementary Fig. 3k, m–p). Altogether, these data indicate that γ2 AMPK activation co-ordinately reduces two fundamental components of the SA cell intrinsic pacemaker mechanism: *I*_f_ and LCRs.

### WGCN analysis links *Prkag2* to a network of pacemaker genes

**Fig. 3:**
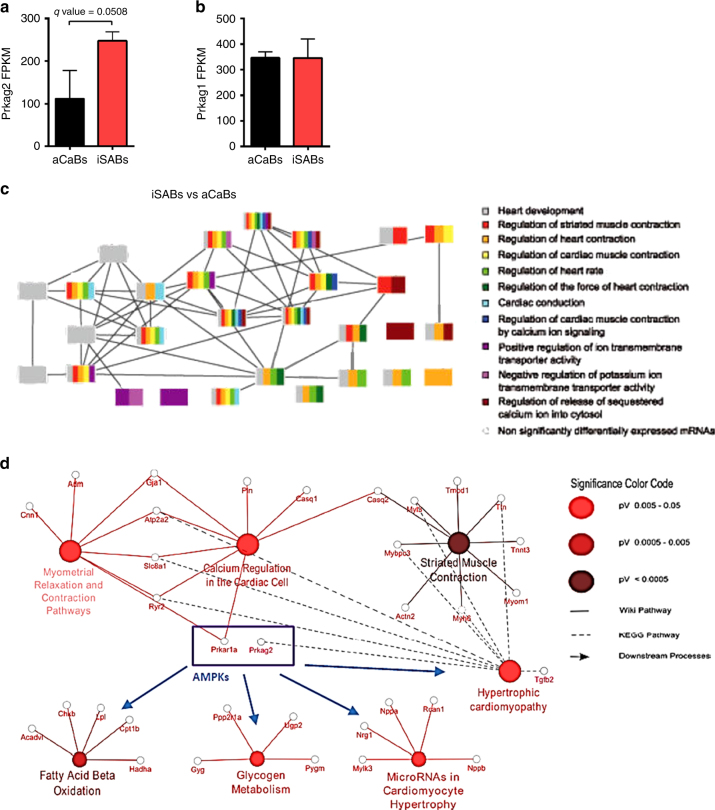
RNA-Seq-derived expression levels of γ AMPK isoforms and gene ontology analysis of iSABs vs aCaBs. **a**, **b** *Prkag2* (**a**) and *Prkag1* (**b**) gene expression in iSABs (induced sinoatrial bodies) vs aCaBs (antibiotic-selected cardiac bodies—a mixture of cardiomyocyte subtypes) by RNA-Seq (*n* = 3). **c**, **d** Functional annotation with gene ontology (GO) analysis of iSAB gene expression identifying enrichment of GO terms associated with pacemaking (**c**) and significant enrichment of AMPK-dependent downstream targets and ontological processes (**d**). **a**, **b** Data are shown as means ± s.e.m.

**Fig. 4:**
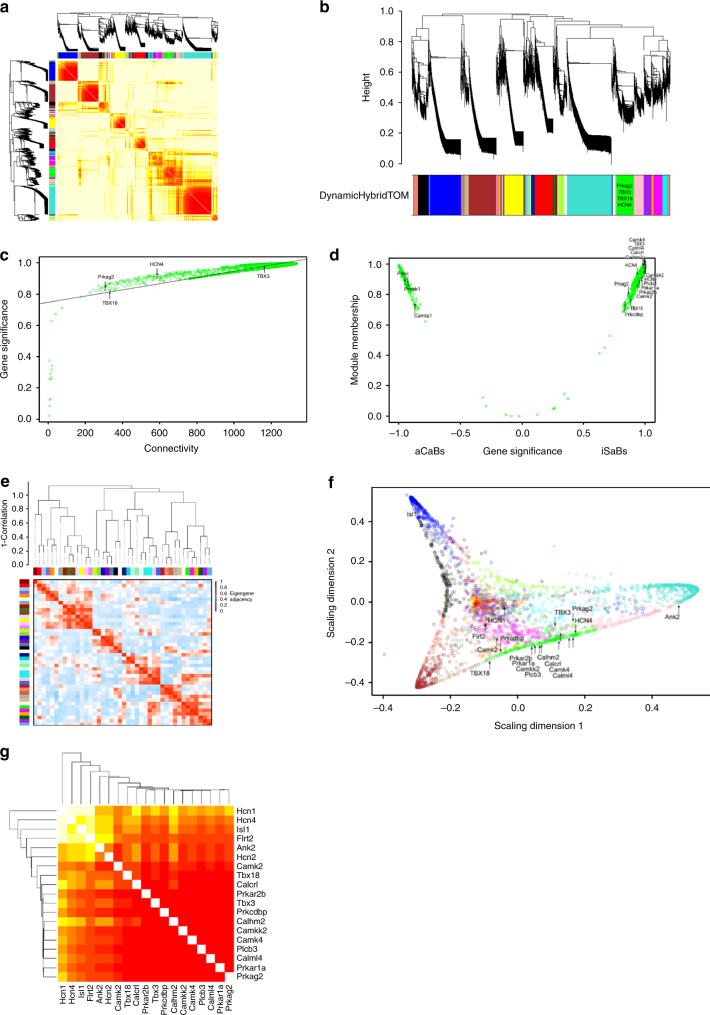
WGCN analysis identifies *Prkag2* in a central hub of pacemaker regulating genes. **a** Weighted gene co-expression network (WGCN) analysis-derived visualization of the iSAB–aCaB gene network by heat map plot. The heat map shows the topological overlap matrix (TOM) among all genes in the analysis. Light yellow represents low overlap and darker red represents higher overlap. Blocks along the diagonal are modules. Dendrograms and module color assignments are shown at the top and along the left side, respectively. **b** Refinement of the gene modules showing the gene dendrogram (average linkage) and module color assignment based on dynamic hybrid TOM clustering. **c** Plot of gene significance and intra-modular connectivity illustrating high correlation within the green module containing *Prkag2*. **d** Plot of co-expressed genes in the green module vs gene significance subdividing iSABs and aCaBs. **e** Further investigation of the relationship and connectivity among the investigated modules illustrated by (upper portion) a hierarchical clustering dendrogram (average linkage) and (lower portion) eigenvalue adjacency heatmap. **f** Multi-dimensional scaling plot identifying the green module as a major signaling hub connecting multiple genes critical to pacemaker functionality. **g** Heat map illustrating the TOM among genes depicted in **e**. Each column and row refers to a single gene. Light yellow represents low overlap and darker red represents higher overlap

**Fig. 5:**
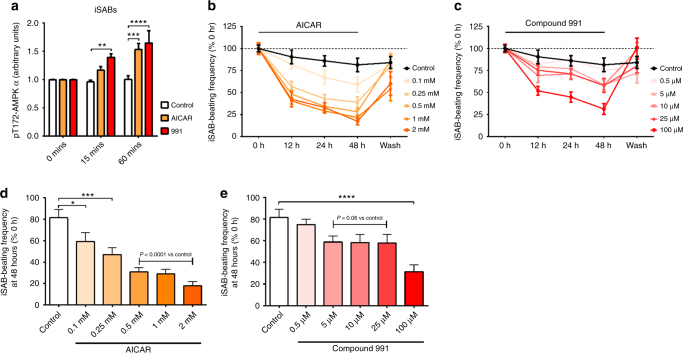
Pharmacological activation of AMPK reduces the spontaneous beating rate of iSABs. **a** ELISA analysis of α AMPK Thr172 phosphorylation in iSABs treated with the AMPK activator AICAR- or the small-molecule AMPK activator compound 991 (*n* = 4). **b** Effect of incubation with variable doses of AICAR or control on iSAB-beating rate. **c** Effect of incubation with variable doses of compound 991 or control on iSAB-beating rate. **d** Bar chart representation of AICAR dose–response effect data shown in **b** specifically for the 48 h incubation time point. **e** Bar chart representation of compound 991 dose–response effect data shown in **c** specifically for the 48 h incubation time point. **a** Two-way ANOVA was performed. **d**, **e** One-way ANOVA followed by Holm–Sidak’s multiple comparisons test was performed. *P < 0.05, **P < 0.01,***P < 0.001, ****P < 0.0001. **a**–**e** Data are shown as means ± s.e.m.

To confirm AMPK’s ability to acutely and reversibly alter intrinsic SA nodal automaticity free from the potential confounding of a constitutive transgenic setting, we first examined the role of AMPK and its modulation using induced murine pacemaker cell aggregates: terminally differentiated induced sinoatrial bodies (iSABs). These are highly pure, spontaneously contracting aggregates consisting substantially of physiologically functional pacemaker cells derived through forward programming with the nodal inducer TBX3 and *Myh6*-promoter-based antibiotic selection of murine pluripotent stem cells^27,28^. Sequencing (RNASeq) of iSABs’ gene expression profiles, when compared to control antibiotic-selected cardiac bodies (aCaBs, a heterogeneous mixture of cardiomyocyte subtypes)^27^, revealed increased expression of γ2, but not γ1 transcript, and significant enrichment of gene ontologies related to AMPK-mediated and Ca^2+^-mediated signaling, the type 1A regulatory subunit of PKA (*Prkar1a*), striated muscle contraction, fatty acid β-oxidation, and glycogen metabolism (Fig. 3a–d). We then constructed a weighted gene co-expression network (WGCN) and identified *Prkag2* in a module (green) of the most highly interconnected genes (corr = 0.74), including *Tbx3*, *Tbx18*, *Hcn4*, *Prkar1a*, *Prkar2b*, *Camk2*, *Camkk2*, *Camk4*, and *Calml4* (Fig. 4a–c). Comparison of iSABs and aCaBs within the module (1,500 and 200 genes, respectively) confirmed that genes expected to be co-expressed in endogenous pacemaker cells were found only in iSABs (Fig. 4d). Hierarchical clustering and multi-dimensional scaling revealed, in commonality with the SA node transcriptome, that the *Prkag2*-containing module represents a major signaling hub with significant connectivity to genes critical to SA node pacemaker function, including *Tbx3*, *Isl1*, and *Hcn4* (Fig. 4e–g). Having identified significant co-expression and correlation of *Prkag2* with known pacemaker-relevant factors in iSABs (Supplementary Table 4), we tested whether pharmacological activation of AMPK (Fig. 5a) could lower iSAB-beating frequency. We observed a reversible, dose–response reduction in beating rate using both an AMP-mimetic agent (AICAR) and a small-molecule cyclic benzimidazole derivative (compound 991), the latter binding specifically to the β subunit of AMPK to cause direct allosteric activation^29,30^ (Fig. 5b–e; Supplementary Movies 1 and 2).

### Adenovirus-mediated γ2 AMPK gain-of-function reduces SA cell firing

**Fig. 6:**
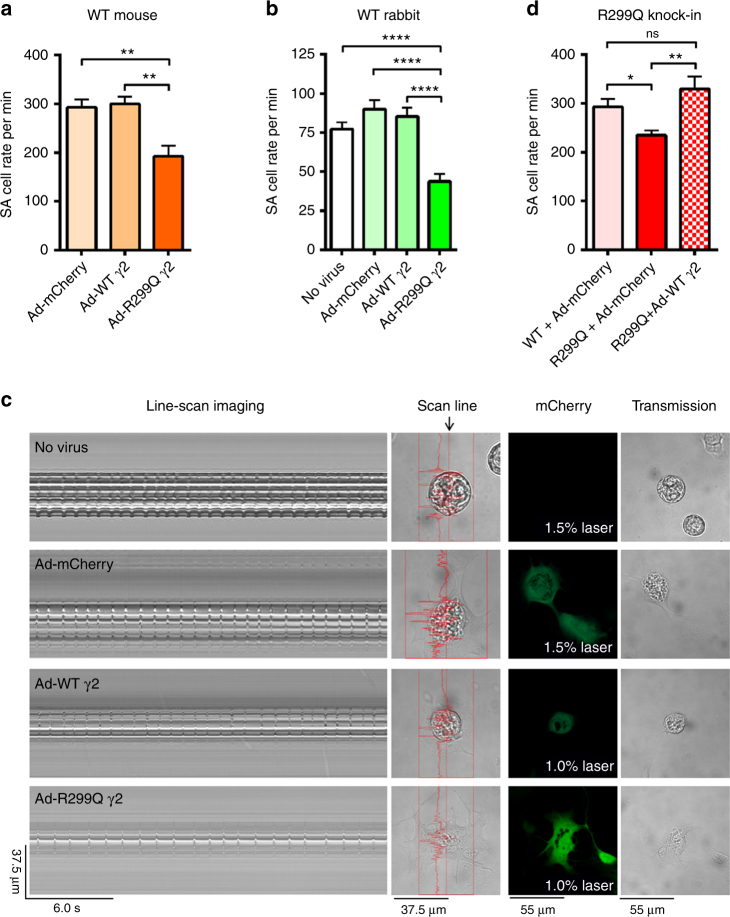
Adenovirus-mediated γ2 AMPK gain-of-function reduces intrinsic firing rate of WT mammalian SA cells. **a** Mean firing rates of SA cells isolated from WT C57BL/6J mice following infection with adenoviruses carrying control (Ad-mCherry), WT γ2 (Ad-WT γ2), or R299Q γ2 (Ad-R299Q γ2) constructs (*n* = 7–9). **b** Mean spontaneous beating rate of WT rabbit SA cells following adenoviral infection (*n* = 29–36). **c** Representative line-scan images of spontaneous contraction (column 1), scan line (column 2), mCherry density (column 3), and corresponding transmission images (column 4) of WT rabbit SA cells following adenoviral infection. **d** Mean firing rates of SA cells isolated from homozygous R299Q γ2 mice following adenoviral infection with control (Ad-mCherry) or WT γ2 constructs (*n* = 5–9). Mean firing rate of WT SA cells following infection with Ad-mCherry (bar identical to that in **a**) also depicted for comparison. **a**, **b**, **d** One-way ANOVA followed by Holm–Sidak’s multiple comparisons test was performed. *P < 0.05, **P < 0.01, ***P < 0.001, ****P < 0.0001; ns not significant. **a**, **b**, **d** Data are shown as means ± s.e.m

We next tested whether acute γ2 AMPK-specific activation could alter the firing rate of individual fully differentiated mammalian WT SA cells. Given the absence of γ isoform-selective AMPK activators, we used adenoviral gene transfer to acutely overexpress R299Q γ2 (Ad-R299Q γ2), WT γ2 (Ad-WT γ2), or empty vector (Ad-mCherry) in primary SA cells isolated from WT (adult C57BL/6J) mice^31^. Whole-cell current-clamp recordings from cultured C57BL/6J SA cells infected with Ad-WT γ2 revealed no detectable effect on spontaneous SA cell beating rate of WT γ2 overexpression (300 ± 15 bpm) over that of Ad-mCherry (293 ± 16 bpm) (Fig. 6a). In contrast, C57BL/6J SA cells infected with adenovirus carrying the activating R299Q γ2 mutation displayed a significantly slower (>30%) beating rate (192 ± 22 bpm, *P* < 0.01 compared with Ad-mCherry or Ad-WT γ2, one-way ANOVA; Fig. 6a). We then asked whether these findings were consistent in a larger mammalian species with electrophysiological properties closer to humans. Adult rabbit SA cells are recognized as excellent models for studying pacemaker mechanisms and exhibit action potentials with significantly closer fidelity to human than rodent species^32^. Congruent with our observations in WT murine SA cells, adenoviral transduction of stable cultured (72 h) adult rabbit SA cells with Ad-R299Q γ2 significantly reduced spontaneous cell beating rate to ~50% of that observed with either empty vector or Ad-WT γ2 (*P* < 0.0001, one-way ANOVA; Fig. 6b, c).

We next applied a similar gene transfer approach in an attempt to rescue the reduced beating rate phenotype of SA cells isolated from homozygous R299Q γ2 mice. Transfection with Ad-WT γ2 vector, through competing out of the mutant allele, completely restored the reduced firing rate of homozygous R299Q γ2 SA cells to that of primary C57BL/6J SA cells treated with empty vector (Fig. 6d).

Collectively, these data establish that (i) acute specific activation of γ2 AMPK is sufficient to substantively reduce the intrinsic firing rate of WT SA cells from two distinct mammalian species, and (ii) the phenotype of reduced SA cell automaticity observed in R299Q γ2 mice can be fully reversed postnatally with short-term gene transfer, arguing against abnormal developmental SA node patterning as a substantial driver of the phenotype in vivo.

### γ2 AMPK has a physiological role in limiting resting HR

**Fig. 7:**
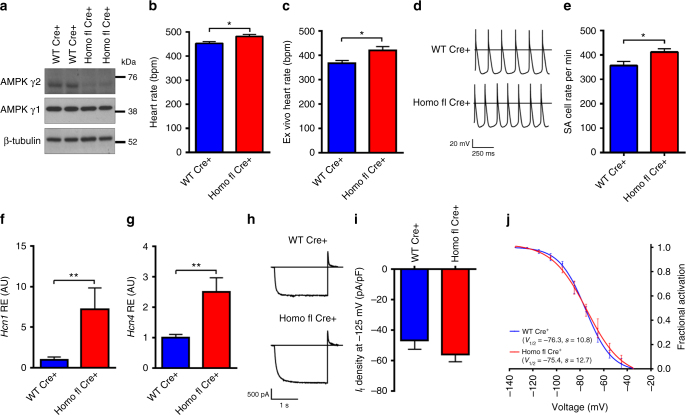
Loss of γ2 AMPK increases resting heart rate and SA cell automaticity. **a** Cardiac western blot for γ AMPK isoforms in Sox2cre-driven γ2 knockout mice (Homo fl Cre+) and WT Sox2cre carrying controls (WT Cre+). **b** HR of Homo fl Cre+ and WT Cre+ mice under anesthesia (*n* = 7–12). **c** HR during ex vivo-isolated cardiac perfusion (*n* = 8–9). **d** Representative action potentials recorded from isolated SA cells. **e** Mean beating rate of SA cells from genotypes illustrated in **d** (*n* = 18–20 cells/3–5 mice). **f**, **g** Relative gene expression (by qRT-PCR) of *Hcn1* and *Hcn4* from whole hearts (*n* = 5–6). **h** Representative *I*_f_ traces during steps to −125 mV. (**i**) Mean *I*_f_ density at −125 mV (*n* = 28–31 cells/7–8 mice). **j** Mean voltage dependence of *I*_f_ activation of isolated SA cells (*n* = 7–9). Uncropped western blots are shown in Supplementary Fig. 10. **b**, **c**, **e**–**g**, **i** Student’s *t*-test was performed. *P < 0.05, **P < 0.01. **b**, **c**, **e**–**g**, **i** Data are shown as means ± s.e.m.

In view of the finding of lower sinus rate associated with the activating R299Q γ2 mutation, we investigated whether tonic γ2 AMPK activation has a physiological role in limiting HR. To address this, we developed a γ2 knockout model by crossing the R299Q γ2 line with Sox2cre transgenic mice to allow global embryonic deletion of the floxed-mutated exon 7 of *Prkag2* (Supplementary Fig. 8a). We confirmed absence of R299Q γ2 transcript, and loss of γ2 AMPK protein and activity, without significant effect on γ1 in these mice (Homo fl Cre+) (Fig. 7a; Supplementary Fig. 8b–f). We also observed no differences in gross cardiac structural or functional phenotype compared with WT Cre+ controls (Supplementary Fig. 8g–j). In contrast to R299Q γ2 mice, and supporting a physiological role for γ2 AMPK activation in regulating HR, we found that γ2 loss led to small but significant increases in HR, both in vivo under anesthesia (Fig. 7b) and during ambulatory telemetric recordings (Supplementary Fig. 9a, b), as well as ex vivo (Fig. 7c). Consistent with greater intrinsic HR, SA cells from Homo fl Cre+ mice displayed enhanced automaticity (Fig. 7d, e), but equivalent MDP and cell capacitance to WT Cre+ (Supplementary Fig. 9c, d). Homo fl Cre+ mice displayed greater cardiac *Hcn1* and *Hcn4* expression than WT Cre+ (Fig. 7f, g); however, we did not identify a statistically significant increase in *I*_f_ density from Homo fl Cre+ isolated SA cells or changes in fractional activation (Fig. 7h–j). In response to isoproterenol, SA cells from Homo fl Cre+ mice reached a similar maximal rate to WT Cre+ but, having starting from a higher baseline rate, reflected a smaller proportional increase from baseline (Supplementary Fig. 9e). SA cells from both genotypes exhibited marked reductions in beating rate in response to acetylcholine, and similar mean shifts in the *I*_f_ activation curve following isoproterenol or acetylcholine stimulation (Supplementary Fig. 9f–h). Measurement of SA cell LCR revealed non-significant trends to greater spontaneous Ca^2+^ signals in Homo fl Cre+ mice compared with WT Cre+ (Supplementary Fig. 9i–m).

### Loss of γ2 AMPK rescues bradycardia in FNIP1-deficient mice

Given the relatively subtle increment in sinus HR observed in the γ2 AMPK knockout, we considered whether the impact of γ2 loss may be more clearly substantiated in a model characterized by severe sinus bradycardia that was likely to be AMPK-dependent. FNIP1 (encoding folliculin-interacting protein 1) is a negative modulator of AMPK. As such it represents an alternative genetic strategy to powerfully activate AMPK. FNIP1 homozygous null mice, through activating AMPK, manifest marked sinus bradycardia^33^. We generated mice deficient in both FNIP1 and γ2 AMPK by crossing FNIP1 null mice with Sox2cre-driven γ2 knockout mice and found that loss of γ2 AMPK was sufficient to rescue FNIP1-deficient bradycardia (Supplementary Fig. 9n). This observation reinforces the conclusions that AMPK activation is sufficient to cause sinus bradycardia, and that the HR effect is directly attributable to the γ2 subunit.

### γ2 AMPK is required to develop intrinsic resting bradycardia of endurance exercise

**Fig. 8:**
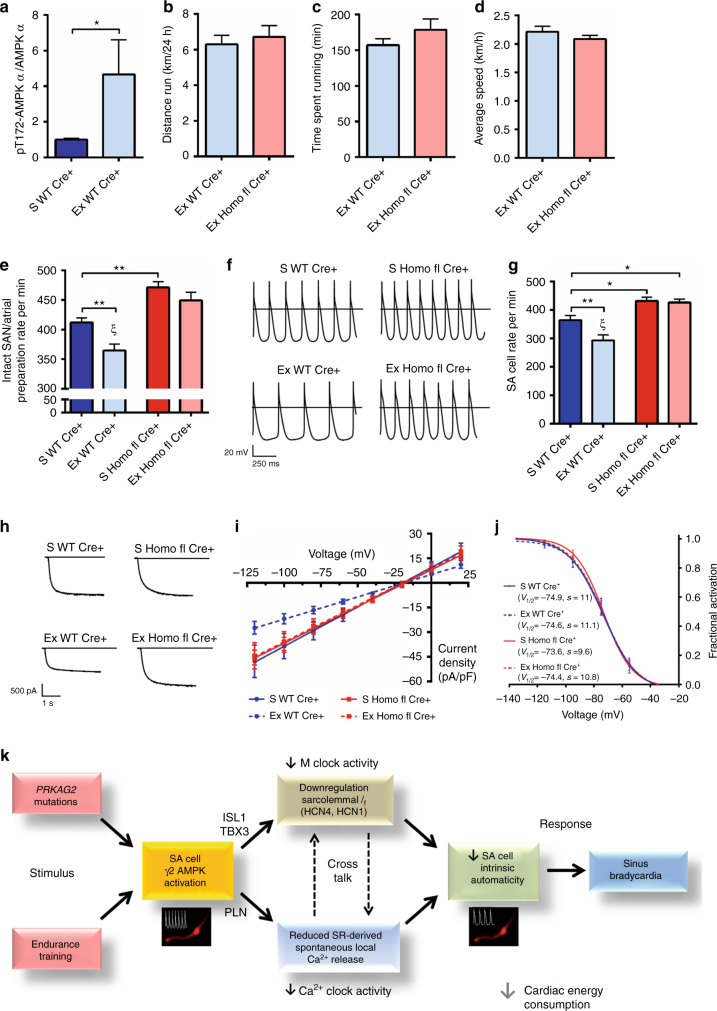
γ2 AMPK is critically required for the intrinsic bradycardic adaptation to endurance exercise. **a** Results of western blot analysis of α AMPK Thr172 phosphorylation in whole heart tissue from sedentary (S) and exercised (Ex, 10 weeks of voluntary wheel running) WT Cre+ mice (*n* = 8–10). **b**–**d** Average daily distance (**b**), time (**c**), and speed (**d**) of voluntary wheel running during a 10–week training period of WT Cre+ and Homo fl Cre+ mice (*n* = 17–26). **e** Spontaneous beating rate of isolated intact SA node/atrial preparations from S and Ex WT Cre+ and Homo fl Cre+ mice (*n* = 10–22). **f** Representative action potentials recorded from isolated SA cells. **g** Mean beating rate of isolated SA cells from S and Ex groups (*n* = 12–27 cells). **h** Representative SA cell *I*_f_ traces during steps to −125 mV. **i** Mean fully activated *I*/*V* curves recorded in SA cells. Linear data fitting yielded statistically significant differences (*P* < 0.0001) in *I*_f_ slope conductance of SA cells from exercised WT Cre+ mice only, with conductance values of 481 (S WT Cre+), 447 (S Homo fl Cre+), 272 (Ex WT Cre+) and 447 pS/pF (Ex Homo fl Cre+) (*n* = 6–14 cells/4–8 mice per group). **j** Mean voltage-dependence of *I*_f_ activation of SA cells from both S and Ex groups (*n* = 6–15). **k** Schematic depicting the central function of SA cell γ2 AMPK in overall cardiac energy accounting. **a**–**d** Student’s *t*-test was performed; **e**, **g**, one-way ANOVA followed by (**e**) Holm–Sidak’s multiple comparisons test or (**g**) Fisher’s least significant difference test was performed. *P < 0.05, **P < 0.01, ^ξ^ *P* < 0.0001 for both Ex WT Cre+ vs S Homo fl Cre+ and Ex WT Cre+ vs Ex Homo fl Cre+ comparisons. **a**–**e**, **g** Data are shown as means ± s.e.m.

To determine whether γ2 AMPK has a broader role in physiological HR regulation, we investigated its involvement in the widely recognized phenomenon of intrinsic resting bradycardia, which follows endurance exercise training^34^. Exercise is known to activate AMPK in skeletal muscle^35,36^, with AMPK in turn having a major role in this tissue’s adaptive response. We examined the effect of 10 weeks of voluntary wheel running exercise (Ex), sufficient to activate cardiac AMPK in resting mice after exercise training (Fig. 8a), on intrinsic HR in comparison to sedentary controls (S). WT Cre+ and Homo fl Cre+ mice ran comparable distances (6.30±0.51 vs 6.72±0.64 km/24 h, *P* = 0.58), durations, and average speeds (Fig. 8b–d). We determined intrinsic HR using ex vivo intact SA node/atrial preparations^37^ and found those of Ex WT Cre+ to display a significantly lower spontaneous beating rate than those from S WT Cre+ mice (364 vs 412 per min, *P* < 0.01, one-way ANOVA)—consistent with training-induced intrinsic resting bradycardia; however, critically, no corresponding training effect was observed on the intrinsic atrial rate of Homo fl Cre+ mice (Fig. 8e). We confirmed these findings in isolated SA cells, observing reduced automaticity of SA cells from trained WT Cre+ but not from trained Homo fl Cre+ mice (Fig. 8f, g). Consistent with a previous report^38^, we observed a clear reduction in SA whole-cell *I*_f_ density in trained WT Cre+ mice, but no effect of training on Homo fl Cre+ mice or fractional activation (Fig. 8h–j). Altogether, these findings indicate a critical gatekeeper function for γ2 AMPK activation to enable the intrinsic bradycardic adaptation to endurance exercise.

## Discussion

By characterizing a murine model of a human AMPK-dependent monogenic bradycardic disorder, we identify a crucial function for γ2 AMPK, traditionally regarded as a minority AMPK subunit, as a major SA isoform with a role in regulating SA node automaticity, and thereby resting HR. This effect is mediated through influencing the major signaling networks of SA cell-autonomous factors regulating pacemaker functionality (e.g., TBX3 and ISL1) and core sarcolemmal (*I*_f_) and subcellular (SR-derived LCRs)-coupled pacemaker mechanisms (Fig. 8k). We observe an opposite HR phenotype resulting from the loss of γ2 AMPK, and describe an indispensable role for this energy sensor in the genesis of intrinsic endurance bradycardia, implicating a non-redundant function for AMPK in mammalian physiological HR regulation and exercise adaptation. The relatively subtle impact of γ2 AMPK loss at baseline, but its obligatory requirement to develop intrinsic endurance bradycardia, exemplify AMPK’s primary function as a sensor that is quiescent at rest but exquisitely responsive to stress.

The R299Q γ2 AMPK gene-targeted mouse model is notable for its relatively restricted cardiac phenotype—contrasting with both the uniformly malignant phenotype of transgenic mice overexpressing mutant human γ2 under a powerful cardiac-restricted promoter and the full expression of the human phenotype—specifically the lack of ventricular pre-excitation or significant LVH. Human PRKAG2 cardiomyopathy is now recognized to be highly heterogeneous, variably penetrant, and generally milder than initially reported, an observation typical of how our understanding of monogenic disorders evolves. We have previously evaluated a series of 20 patients with the R302Q *PRKAG2* mutation, orthologous to the knock-in mutation carried by R299Q γ2 knock-in mice. Although 18 of these patients (90%) had sinus bradycardia, only 2 (10%) had LVH and none had WPW syndrome. Other clinical reports document the absence of LVH with this or other *PRKAG2* mutations^39,40^, suggesting that neither LVH nor pre-excitation are universal features of human PRKAG2 cardiomyopathy.

The phenotype of transgenic mice often differ from humans. In addition to recognized functional differences between mice and humans in terms of allometric scaling, prominent in the cardiovascular system and predisposing humans to a more marked bradycardia *per* se, an additional difference accounting for the subtlety of the heterozygote (and indeed homozygote) murine phenotype is likely to be the relative difference in the cardiac expression of γ2 in mouse vs human. Use of more penetrant mutations and an overexpression transgenic approach are likely to be required to consistently generate the more extreme end of the phenotypic spectrum in mice. Substantiating these gene dosage and stoichiometric considerations, other recently generated gene-targeted γ2 AMPK-mutant mice bearing mutations with severe biochemical consequences exhibit a remarkably consistent sinus bradycardia but otherwise subtle cardiac phenotype^41^.

AMPK, by virtue of being at the intersection of systemic energy sensing and caloric regulation^9^, appears well placed to determine the established coupling between basal metabolic rate and HR, and is likely to contribute to various allometric scaling phenomena, which seem to have empirical validity, albeit imperfect, over a diverse range of organisms^42^. Activation of γ2 AMPK complexes remodels SA cell gene expression and electrophysiology, reducing intrinsic resting HR to diminish myocardial work. A central inference of our study is that, with its co-option at the metazoan divergence, AMPK has transitioned from being a purely cell-autonomous regulator of energy charge to co-ordinating organ-specific and systemic-caloric accounting. An example of this broader influence is its indispensable role in the regulation of exercise-related changes in HR.

Akin to the similarly highly conserved clock genes that regulate circadian biology at multiple hierarchical levels, ranging from cell-autonomous regulation, through entrainment of organ-specific time cues at different developmental stages, to the systemic control of both the persistence and periodicity of circadian rhythms^43^, γ2 AMPK regulates the firing frequency of individual pacemaker cells, thereby gating basal cardiac contractile rate to ensure medium- to long-term myocardial energy homeostasis and to influence whole-mammal energy expenditure^9^.

Athletes’ hearts need to remain parsimonious at rest, yet retain the capacity to perform optimally during vigorous physical activity. In the context of an increased stroke volume due to cardiac chamber enlargement, a reduced intrinsic sinus rate—mediated by intermittent physiologic SA node γ2 AMPK activation—maintains basal cardiac energy expenditure, yet leaves substantial chronotropic reserve to accommodate peak activity demands; loss of γ2 AMPK specifically abrogates this adaptation. In normal physiology, SA node γ2 AMPK activation acts as a brake to chronically increased HR, mitigating against substantial cardiac energy expenditure. Conversely, pathological *PRKAG2* mutations result in inappropriate and persistent elevation in SA node γ2 AMPK activity, effectively tonically activating the signal driving the intrinsic resting bradycardic response to endurance exercise. In a bidirectional way, therefore, our findings explain the deleterious SA node pathology observed in *PRKAG2* mutation carriers and provide a molecular substrate for increasingly recognized, albeit infrequent, potential long-term sequelae of the athletic heart, which can include increased risk of symptomatic SA node disease with need for pacemaker implantation in later life^44^. The specific ability of γ2-containing AMPK complexes to regulate intrinsic SA node firing and HR raise the possibility that its selective modulation may hold therapeutic potential in both states.

## Methods

### Human R302Q γ2 heterozygous carriers and HR measurement

Assessment of HR in human subjects was approved by the local Research Ethics Committee (Comitê de Ética em Pesquisa, Faculdade Ciências Médicas, Minas Gerais, Brazil). All study subjects provided written informed consent prior to participation. All subjects underwent genotyping for the R302Q γ2 mutation by PCR amplification and fluorescent dideoxy sequencing of exon 7 of *PRKAG2*. Mean HR was obtained from 15 heterozygous R302Q γ2 carriers and 10 genotype-negative sibling controls (41.2 ± 2.8 vs 38.9 ± 2.3 years, mean ± s.e.m.) using 24-h HR monitors (DMS Cardioscan Premier 11 Recorder DMS 300-8). In subjects with indwelling permanent pacemakers (6 R302Q γ2 carriers and no controls), HR was assessed by programming the generator to VVI mode and measuring the HR after a waiting period of 10 min. Anti-arrhythmic drugs (including β-blockers) were discontinued for at least 5 days before HR assessment. No subject had atrial fibrillation or was on amiodarone.

### Animals

Animal studies were performed in accordance with the 1986 British Home Office Animals Scientific Procedures Act (UK) incorporating European Directive 2010/63/EU, the European Directive (86/609/CEE) on the care and use of laboratory animals, and the Guide for the Care and Use of Laboratory Animals published by the National Institutes of Health (NIH Publication no. 85–23, revised 1996). All experimental protocols involving animals were assessed and approved by the local ethical review committee: University of Oxford Animal Welfare and Ethical Review Body; Animal Welfare committee of the University of Milan and the Italian Minister of Health (Italian D.lgs 116/92 and D. Lgs no. 2014/26); the NIH Institutional Animal Care and Use Committee; and the University of Colorado Denver—Anschutz Medical Campus Institutional Animal Care and Use Committee (protocol number 84814(06)1E). Experimental animal work was undertaken blind to genotype.

R299Q γ2 mice have been previously described^9^. Gene targeting was used to introduce the R299Q point mutation (equivalent to the human R302Q mutation) into exon 7 of murine *Prkag2*. The gene-targeting vector contained a shorter 5′ homology arm (in intron 6 and exon 7) amplified by PCR from genomic DNA isolated from 129Sv embryonic stem (ES) cells. The point mutation was introduced by PCR and the positive selection cassette (*neo*), flanked by FRT (Flp recombinase recognition target) sites to enable excision of the neo cassette, was inserted in front of exon 7. The targeting strategy included insertion of *loxP* sites within intron 6 upstream and within intron 7 downstream of the mutation, respectively. The longer 3′ homology arm was obtained from a BAC clone (Sanger Institute, Cambridge, UK), with a Diphtheria Toxin A (DTA) cassette attached to the 3′ homology arm for negative selection. The homology arms with the mutation were cloned into a suitable targeting vector in our laboratory; selection cassettes and genomic DNA were provided by GenOway (Lyon, France). Transfection of 129Sv embryonic stem (ES) cells, selection, isolation and confirmation of positive clones by Southern blot analysis, injection of positive clones into C57BL/6 embryos and in vivo excision of the positive selection cassette were performed by GenOway. Correct homologous recombination in the positive ES clones was confirmed by Southern blot analysis. The presence of the point mutation and the distal *loxP* site were validated by sequencing. Positive clones were injected into C57BL/6 embryos. Highly chimeric males were then bred with Flp-expressing mice and the *FRT*-flanked neo cassette deleted, resulting in a floxed knockin *Prkag2* allele. Mice heterozygous for the R299Q γ2 knockin mutation were initially on a mixed C57BL/6/129/Ola genetic background and subsequently backcrossed to C57BL/6 for at least seven generations. R299Q γ2 mice were genotyped by PCR from earnotch tissue-derived gDNA using primers (Supplementary Table 5) hybridizing either side of the *loxP-FRT* sequence in intron 6 to distinguish the WT *Prkag2* locus from the recombined, Flp-excised *Prkag2* allele.

Sox2cre transgenic mice^45^ that had been backcrossed for six generations onto a C57BL/6 genetic background were crossed with R299Q γ2 mice to achieve global γ2 deletion via the conditional deletion of the floxed exon 7 of *Prkag2*. Mice deficient in both alleles, representing a global knockout of γ2 (termed Homo fl Cre+), were compared with control mice hemizygous for the Sox2cre transgene but wild-type for *Prkag2* (termed WT Cre+). Sox2cre γ2 knockout mice were genotyped from gDNA using separate PCRs to assess for the R299Q γ2 mutation (as above), the Sox2cre transgene, and detection of intact and excised exon 7 from *Prkag2* (primer sequences detailed in Supplementary Table 5).

The generation and phenotype of *Fnip1* null mutants is as previously described^33^ (MGI ID:5806459).

### Allelic discrimination

Competitive multiplex PCR of cardiac cDNA for specific detection of R299Q γ2 transcript was undertaken using TaqMan MGB fluorogenic probes specific for the wild-type (5′-FAM-AGTCCGTGCAGCGC-MGB-3′) or mutant (5′-VIC-AGTCCAAGCAGCGC-MGB-3′) *Prkag2* sequence and common exon-spanning primers (Supplementary Table 5) flanking the site of the mutation on exon 7. Primers were designed (Primer Express 3.0) and reactions were undertaken in accordance with published guidance^46^ on a StepOne Real-Time PCR system (Applied Biosystems). Data analysis and visualization were with StepOne software (v2, Applied Biosystems).

### Western blotting

Protein extraction and western blotting were undertaken largely as previously described^47^. Briefly, snap-frozen cardiac tissue aliquots were ground in liquid nitrogen and homogenized in ice-cold buffer comprising 50 mM Tris base, 250 mM sucrose, 1 mM EDTA, protease and phosphatase inhibitor cocktail tablets (Roche, West Sussex, UK), 50 mM NaF, 5 mM sodium pyrophosphate, 1 mM dithiothreitol (DTT), 1 mM benzamidine, 0.1 mM phenylmethylsulphonyl fluoride (PMSF), and 1 mM sodium orthovanadate. Extracts were then centrifuged at 13,000×*g* for 15 min at 4 °C. Protein concentration was determined from diluted aliquots of the soluble fraction by BCA protein assay (Thermo Fisher Scientific) with samples then diluted in fresh lysis buffer to yield equivalent final protein concentrations. Lysates were mixed with lithium dodecyl sulfate sample buffer with DTT (50 mM) (nuPAGE, Invitrogen) and boiled at 95 °C for 5 min. For western blotting of SA nodes, each sample was pooled from three individual SA nodes and homogenized and lysed in RIPA buffer (Thermo Fisher Scientific: 25 mM Tris-HCl (pH 7.6), 150 mM NaCl, 1% NP-40, 1% sodium deoxycholate, 0.1% SDS) supplemented with Halt protease inhibitor cocktail (Thermo Fisher Scientific), Halt phosphatase inhibitor cocktail (Thermo Fisher Scientific) and 1 mM phenylmethyl sulfonyl fluoride, using a Precellys24 homogenizer (Bertin Instruments) with tissue homogenization kit at 4 °C. Loading controls were run on the same blot.

SDS-PAGE was undertaken on pre-cast polyacrylamide gels (Nupage 4–12% Bis Tris gel, Novex, Invitrogen) and transfered onto polyvinylidene difluoride membranes (Immun-Blot, Bio-Rad) using an electrophoretic transfer cell (Mini Trans-Blot, Bio-Rad). Blocked membranes (5% milk/tris-buffered saline with Tween-20, TBST) were incubated with primary antibody, followed by TBST washes and secondary horseradish peroxidase (HRP)-conjugated antibody detection. Bands were visualized using ECL reagents (GE Healthcare, Buckinghamshire, UK), and films scanned with subsequent analysis of digital images using ImageJ (NIH). Uncropped western blots accompanied by the location of molecular weight markers are shown in Supplementary Fig. 10.

The following antibodies were used: anti-phospho-ACC (#3661) at 1:1,000 working concentration, anti-ACC (#3676) at 1:1,000, anti-phospho-AMPK Thr172 (#2535) at 1:1,000 from Cell Signaling (New England BioLabs, Hertfordshire, UK); anti-γ1 AMPK (ab32508) at 1:1,000 and anti-β-tubulin (ab6046) at 1:4,000 from Abcam (Cambridge, UK); anti-γ2 AMPK (sc-19141) at 1:500 and anti-α2 AMPK (sc-19131) from Santa Cruz Biotechnology (TX, USA); anti-HCN4 (APC-052) at 1:200 from Alomone Labs (Israel); anti-GAPDH (MAB374) at 1:4,000 from Merck Millipore (Hertfordshire, UK); anti-PLN (ab86930) at 1:2,000, anti-SERCA2 ATPase (ab91032) at 1:2,000, anti-NCX1 (ab177952) at 1:2,000 from Abcam (Cambridge, MA, USA); anti-CASQ at 1:2,000 (PA1-913), anti-RYR2 at 1:1,000 (MA3-916) and anti-DHPR1 alpha (for LTCC, PA5-23010) at 1:1,000 from Thermo Fisher Scientific (Waltham, MA, USA). HRP-conjugated secondary antibodies used were anti-rabbit IgG (NA934) from GE Healthcare and anti-goat IgG (ab6741) from Abcam.

### AMPK activity assay

Cardiac AMPK activity was measured from immunoprecipitated AMPK complexes by SAMS peptide phosphorylation assay essentially as previously described^15^. In brief, protein extracts were prepared as per samples for western blotting, including addition of phosphatase inhibitors. AMPK γ subunit isoform-specific antibody was pre-bound to a 50% protein G-sepharose bead slurry on an orbital shaker (IKA Vibrax VXR) at 4 °C for 2 h. These were then gently centrifuged and washed in ice-cold PBS/1% triton, followed by a further ice-cold PBS wash. Tissue lysate was added to pre-bound protein G-antibody mix in ice-cold 1% triton/HBA buffer (50 mM HEPES, 50 mM sodium fluoride, 5 mM sodium pyrophosphate, 1 mM EDTA, 10% glycerol [v/v], 1 mM DTT, 1 mM benzamidine, 0.1 mM PMSF, supplemented with a protease inhibitor cocktail tablet (Roche), pH to 7.4 at room temperature). Immunoprecipitation (IP) was performed on an orbital shaker at 4 °C for 2–3 h (typically IP 30 μL protein G-Sepharose/antibody slurry and 250 μg of sample lysate, made up to 500 μL in HBA/1% triton with fresh protease inhibitors).

In-house antibodies were used for immunoprecipitation of γ2 (rabbit polyclonal, C-terminus directed) and γ1 (rabbit polyclonal) AMPK. AMPK activity from immune complexes was determined by measuring the incorporation of [γ-^32^P]-ATP into the SAMS synthetic peptide substrate, with/without 0.2 mM AMP, by scintillation counting (Tri-Carb 2800TR, PerkinElmer, UK).

### In vivo cine magnetic resonance imaging

High-resolution in vivo cine MRI was performed on a cohort of R299Q γ2 mice and WT littermate controls at 2 and 10 months of age, to accurately assess left ventricular volumes, function, and mass with high spatial resolution. Anesthesia was induced in an anesthetic chamber using 4% isoflurane in 100% oxygen. Electrodes were positioned subcutaneously, and mice were positioned prone on a dedicated mouse cradle and maintained at 1.5–2% isoflurane at 2 L/min oxygen flow. Temperature was maintained at ~37 °C using a homeostatically controlled warm air blanket. Cardiac and respiratory signals were continuously monitored and used for combined ECG gating and respiratory gating. Eyes were protected with a petroleum-based ophthalmic ointment. Cine MRI experiments were carried out using a horizontal 210 mm bore 9.4 T magnet with VNMRS DirectDrive console and 60 mm i.d. 1,000 mT/m actively shielded gradient system (Agilent Technologies, USA). A 33 mm internal diameter, quadrature-driven birdcage resonator (Rapid Biomedical, Germany) was used for signal transmission/reception. Cine imaging was carried out as described previously^48^. Multi-frame left ventricular-short axis slices were acquired (7–10 contiguous slices, 1 mm thickness, 18–32 frames per cardiac cycle) covering apex to base. Images were reconstructed off-line as TIFF files using custom-written software. End-diastolic and end-systolic frames were selected for each slice according to maximal and minimal ventricular cavity size and semi-automated image segmentation performed by a single operator using AMIRA software (Visage Imaging) blind to mouse ID/genotype.

### In vivo cardiac ^31^P magnetic resonance spectroscopy

MR spectroscopy experiments were carried out using a 9.4 T magnet as above with 120 mm i.d. 600 mT/m actively shielded gradient system (Agilent Technologies, USA). An actively decoupled variable tune/match 14 mm diameter^31^P surface coil was purpose built in-house and used in conjunction with a double tuned ^1^H/^31^P volume resonator (Rapid Biomedical, Germany) for acquisition. Animals were prepared as described above. Shimming and scouting were carried out using the^1^H channel of the volume coil. A removable 4 mm point sphere filled with 15 M H_3_PO_4_ was placed outside the animal cradle to allow for accurate and rapid pulse calibration using an unlocalized pulse-acquire experiment. 2D acquisition weighted (Hanning) CSI (chemical shift imaging) data were acquired for WT, heterozygote R299Q γ2, and homozygote R299Q γ2 male mice aged ~10 weeks (*n* = 7, 18, 9, respectively) from a 5 mm thick mid-ventricular short axis slice (in-plane voxel size of 2.31 × 2.31 mm, 13 × 13 PE steps, 30 × 30 mm FOV, 5 mm slice thickness, 8191 scans, TE=0.87 ms). Acquisitions were cardiac gated and a TR of ~250 ms (two cardiac cycles) was used with a 30° flip angle. Total scan time for the experiment was ~35 min. Multi-slice ^1^H anatomical images covering the field of view of the CSI experiment were acquired to confirm the position and tissue content of the CSI voxels. Fully sampled data were zerofilled to 64 × 64 PE steps, and reconstructed as described previously^49^.

For each mouse, a 3 × 3 grid of spectra from voxels located at the septum of the heart was fitted using a Voigt lineshape (in-house software), and the PCr, γ-ATP, and 2,3-diphosphoglycerate (DPG) signal amplitudes estimated. The spectrum with the lowest γ-ATP/DPG ratio was selected as the nominal blood spectrum, and its PCr and γ-ATP amplitudes, normalized to DPG amplitude, were subtracted from those of the remaining eight myocardial spectra. Finally, T1 saturation correction for residual PCr and γ-ATP amplitudes was carried out using the mean TR for the CSI experiment, and published T1 values^50^. The mean PCr/γ-ATP ratio was then calculated for the myocardium.

### Cardiac histology

Hearts were rinsed in ice-cold PBS and immerse-fixed in 10% neutral buffered formalin (VWR, Leicestershire, UK) for 24 h, then processed in an automated tissue processor (Bavimed Histomaster, Germany) overnight. Cross-sections (5–7 µm) were obtained using a microtome (Leica RM 2155), spread onto polysine-coated glass slides (VWR), and allowed to dry. Sections were stained with hematoxylin and eosin (Sigma-Aldrich, Dorset, UK), periodic acid-Schiff (Sigma-Aldrich) or Picrosirius red (Polysciences, Germany). Images were acquired with a Nikon light microscope (Nikon Eclipse TE2000U) coupled to a digital camera (Nikon Digital Sight DS-5M).

### Transmission electron microscopy

Hearts were extracted, cut finely into small (~1 mm^3^) blocks and fixed overnight in 4% glutaraldehyde in 100 mM phosphate buffer, followed by post-fixation in 2% osmium tetroxide in 100 mM phosphate buffer. Specimens then underwent en bloc treatment with uranyl acetate, dehydration in ethanol, and transferred to propylene oxide, prior to embedding. Ultra-thin sections (50–70 nm) were cut and stained with uranyl acetate and lead citrate, and examined in a JEOL 1200EX electron microscope.

### Biochemical glycogen content

Cardiac glycogen was quantified in R299Q γ2 and WT mice aged 12 months essentially as described^15^. In brief, snap-frozen tissue samples ground under dry ice were homogenized in lysis buffer (50 mM Tris, 0.25 M mannitol, 1 mM DTT, 0.1 mM PMSF adjusted to pH 7.4 and supplemented with protease inhibitor tablets [Roche]) and sonicated prior to syringe-and-needle homogenization. A total of 2 M KOH at 70 °C was used to solubilize glycogen followed by amyloglucosidase digestion overnight to release glucose. Glucose content was then determined using an enzyme coupled assay (Roche) to generate glucose-6-phosphate (G6P) by hexokinase, followed by oxidation of G6P by G6P dehydrogenase in the presence of NADP^+^, with spectrophotometric measurement of NADPH after 30 min.

### Cardiac qPCR

RNA extraction, cDNA synthesis, and RT-PCR were performed as previously described^47^. qRT-PCR using inventoried TaqMan gene expression assays was used to determine relative gene expression of the following: γ2 and γ1 AMPK in wild-type C57BL/6 murine SA nodes and LVs (*Prkag2*, Mm00513977_m1; *Prkag1*, Mm00450298_g1); and cardiac expression of *Slc2a1* (Mm00441480_m1), *Slc2a4* (Mm00436615_m1), *Gyg* (Mm00516516_m1), and *Pygm* (Mm00478582_m1) in R299Q γ2 and WT mice. Relative expression of the target gene was quantified using the 2^−ΔΔCt^ method using *Actb* (Mm00607939_s1) as the endogenous control (where ΔCt is the difference in cycle threshold value between the target transcript and the endogenous control). Samples were analyzed in at least duplicate, and samples minus reverse transcriptase enzyme and samples minus cDNA template were used as negative controls (to exclude gDNA contamination or reagent cross-contamination, respectively) and checked to ensure they failed to reach threshold by 40 cycles.

### SA node histology

SA nodes were harvested using an operating microscope and 12 consecutive 5 μm paraffin-embedded sections obtained. SA node sections were stained with the following: Masson’s Trichrome (Masson’s Trichrome stain kit, American MasterTech, Lodi, CA); reticulum plus picric red stains to determine collagen and outline atrial myocytes (Chandler’s precision reticulum stain kit, American MasterTech); and Periodic acid-Schiff (PAS kit, American MasterTech). High-resolution digital images of cross-sections were acquired using a Leica microscope. Morphometric evaluation was performed using a computerized imaging analysis system (Metamorph, University Imaging) blind to genotype according to a modified prior method^51^. Quantification of PAS-stained areas and cells (clusters of small, round, homogeneous PAS-positive granular structures) was taken from images at ×400 magnification.

### Invasive electrophysiology

Conduction parameters were assessed in 3-month-old R299Q γ2 and WT littermate controls based on modifications of a published EPS protocol for mice^52^. Under 1.5% isoflurane anesthesia, a 1.1F electrophysiology catheter (EPR-801, Millar Instruments) was introduced into the right atrium and ventricle via the right internal jugular vein. High-fidelity intracardiac electrograms were filtered at 0.5–500 Hz and continuously recorded using PowerLab and LabChart software (ADInstruments, Oxford). After obtaining a baseline surface ECG, programmed stimulation protocols were performed as described^53^ using a pacing stimulator with output set at twice diastolic threshold (s88 Grass stimulator, Grass technologies, USA). Sinus cycle length (SCL) and sinus node recovery time (SNRT) were determined by delivering an atrial pacing train at a cycle length of 100 ms for 15 s. SNRT was calculated as the maximum interval from the last-paced atrial complex to the first spontaneous complex after cessation of pacing. All measurements were repeated in triplicate. Atrioventricular (AV) Wenckebach cycle length (AVW) and AV 2:1 cycle length were determined using burst atrial pacing by progressively reducing pacing cycle length from baseline in decrements of 10 ms (incremental atrial pacing) until 1:1 AV conduction was reliably lost (Wenckebach cycle length), or resulted in failure of conduction of every other pacing stimulus to the ventricle (2:1 cycle length). AV nodal effective refractory period (AVNERP) was determined using programmed atrial extrastimulus pacing with an eight stimuli atrial drive train (S1) delivered at 100 ms, followed by a single premature stimulus (S2) progressively decremented until ventricular conduction was lost. AVNERP was defined as the longest S1–S2 coupling interval where S2 failed to capture the ventricle. Right atrial programmed electrical stimulation was used in an attempt to induce an atrioventricular re-entrant tachycardia (AVRT) as evidence for the existence of an accessory pathway. An atrial double extrastimulus technique was used with an eight stimuli primary drive train (S1) delivered at a fixed cycle length of 100 ms, followed by premature stimuli (S2 and S3) coupled at 80 ms and decremented to refractoriness. Further provocation for AVRT included atrial-burst pacing at fixed cycle length, including following β-adrenoceptor stimulation by isoproterenol administration (1 ng/g IV). All recordings were analyzed off-line, blind to genotype, using LabChart software (ADInstruments).

### ECG biotelemetry

Data were recorded using biotelemetry sensors (HDX-11 or ETA-F20 from Data Sciences International, St. Paul, MN) recorded at 1000 Hz. All recordings were taken during the light cycle with mice held in a standard 12 h light–dark cycle with constant temperature and humidity.

### Ex vivo cardiac perfusion and HR

Ex vivo cardiac perfusion was performed as previously described^47^ with minor modifications. Briefly, following pentobarbitone anesthesia (140 mg/kg IP) and systemic heparinization (150 IU), hearts were removed, cannulated, and retrogradely perfused at 37 °C in Langendorff mode at a constant perfusion pressure of 80 mmHg with a modified Krebs–Henseleit buffer that contained the following (in mmol/L): NaCl, 118.5; NaHCO_3_, 25; KCl, 4.75; KH_2_PO_4_, 1.18; MgSO_4_, 1.19; CaCl_2_, 1.41; d-glucose, 11; pyruvate, 5; pH 7.4; saturated with 95% O_2_ and 5% CO_2_. Inclusion criteria used were: time interval to aortic cannulation of under 3 min and absence of persistent arrhythmias during stabilization. For measurement of AMPK activity in the context of preserved cellular energetic status, hearts were freeze-clamped after a 25 min stabilization period of perfusion as previously described^21^. For ex vivo HR, we used Krebs–Henseleit buffer without supplemental pyruvate. A fluid-filled polyvinylchloride film balloon was introduced into the LV and connected to a pressure transducer (ADInstruments). The reading was digitally processed to provide a ventricular pressure recording from which HR was subsequently derived blinded to genotype. Intrinsic HR was recorded as the average measured rate over 30 s, 5 min after cannulation.

### SA node isolation and RNA extraction

The heart with lungs was quickly removed and immersed at 4 °C to wash out the blood in an external solution that contained the following (in mmol/L): NaCl, 137; KCl, 4.9; NaH_2_PO_4_, 1.2; glucose, 15; HEPES, 20; MgCl_2_, 1; pH 7.4. The heart–lung block was pinned to the tissue bath to excise the right atrium (RA) and SA node under a stereomicroscope. The tissue bath was perfused with the external solution at a rate of 10 mL/min. After removal of both ventricles and the left atrium, the RA was opened to expose the crista terminalis, the intercaval area and the interatrial septum. This preparation was pinned by small stainless steel pins to the chamber with the endocardial side exposed up and trimmed carefully to extract the SA node region correctly. The SA node region was delimited by the borders of the crista terminalis, the interatrial septum, superior and inferior vena cava. All tissues were snap-frozen in liquid nitrogen and stored at −80 °C for subsequent RNA extraction. Four mouse SA node tissues were pooled and processed together for the extraction of one sample of total RNA. RNA was extracted with an RNeasy Mini Kit (Qiagen) using DNase on-column digestion according to the manufacturer’s protocol.

### SA node gene expression profiling and microarray analysis

R299Q γ2 and WT littermate controls (four homozygote R299Q γ2, four heterozygote R299Q γ2, and three WT) were hybridized to Illumina MouseRef-8 v2 bead chips. BeadArrays were scanned by the Illumina BeadStation 500X. All data were log_2_-transformed and normalized by Robust Spline Normalization using the lumi software package in Bioconductor^54^. Overall, 18,185 nucleotide probes were filtered from 25,697 total using an Illumina detection *P* value of *α* = 0.05. Significant genes were selected by a one-way ANOVA or *t*-test (FDR = 0.05) for further analysis. GSEA software^55^ was used to select differentially expressed (DE) genes by calculating out a “score” using the Kolmogorov–Smirnov (KS) test. The score indicates how a gene relates with genotype, where positive values relate to upregulation and negative values relate to downregulation. Only transcripts with a KS score > |0.15| were considered DE. Transcripts demonstrating greater than 1.2-fold change in expression were processed using Ingenuity Pathway Analysis software (Ingenuity Systems Inc., CA, USA) to identify networks and canonical pathways overrepresented in enriched genes.

### SA node microarray validation

Total RNA (850 ng) were used for cDNA preparation in a 40 µL reaction volume with MMLV reverse transcriptase (RT, Life Technologies, CA, USA) with random hexamers. All cDNA synthesis reactions were accompanied by no template controls for the detection of possible contamination and no RT controls to detect potential genomic DNA. qRT-PCR was performed using an ABI Prism 7900HT Sequence Detection System (Applied Biosystems) with a 384-well platform. Reactions were performed with FastStart Universal SYBR Green Master mix with ROX (Roche) using manufacturer recommended conditions. Forward and reverse primers used are detailed in Supplementary Table 5.

Preliminary reactions were run to determine amplification efficiency. The size of the amplicon and its specificity were assessed by agarose gel analysis and a post-amplification dissociation curve, respectively. Each well contained 0.5 µL of cDNA solution and 10 µL of reaction mixture. Samples were assessed in quadruplicate and relative expression calculated using the 2^−ΔΔCt^ method using *Hprt* as endogenous control (where ΔCt is the difference in cycle threshold value between the target transcript and the endogenous control). Statistical analysis was undertaken using a one-tailed Student’s *t*-test.

### Murine SA cell electrophysiology and *I*_f_ measurement

Hearts were extracted, and the SA node region dissected and used for isolation of SA cells by an enzymatic and mechanical dissociation procedure as previously described^56^. Briefly, hearts were rapidly removed and placed in a pre-warmed (37 °C) Tyrode solution (in mmol/L: NaCl, 140; KCl, 5.4; CaCl2, 1.8; MgCl2, 1; d-glucose, 5.5; HEPES-NaOH, 5; pH 7.4) containing 1,000 U heparin. The SA node region was surgically exposed, isolated, and cut into small strips, which were placed in an enzymatic solution containing the following: collagenase (224 U/ml, Worthington), elastase (1.42 U/mL, Sigma-Aldrich), and protease (0.45 U/mL, Sigma-Aldrich) to loosen intercellular connections. The enzymatic solution was then removed and SA strips placed in a high-K^+^, low-Na^+^, Ca2^+^, and Mg^2+^-free solution. Cells were then fully dispersed and separated by manual agitation of SA strips using a glass pipette with a small tip (~2–3 mm diameter). Cells were finally reacclimatized to physiological concentrations of NaCl, KCl, MgCl_2_, and CaCl_2_ by adding the necessary amounts of a solution containing 1.8 mM CaCl_2_ and 10 mM NaCl, and normal Tyrode with BSA 1 mg/mL. Isolated single cells were kept at 4 °C for the day of the experiment and patch-clamp experiments were performed in the whole-cell configuration at 35 ± 0.5 °C. The pipette solution contained (in mmol/L): K-aspartate, 130; NaCl, 10; EGTA-KOH, 5; CaCl_2_, 2; MgCl_2_, 2; ATP (Na-salt), 2; creatine phosphate, 5; GTP (Na-salt), 0.1; pH 7.2. Action potentials were recorded from spontaneously beating SA node myocytes or small uniformly beating aggregates of pacing cells (2–5 cells) superfused with normal Tyrode solution and the rate and MDP values measured with customized software. *I*_f_ was recorded from single cells superfused with Tyrode solution containing BaCl_2_ (1 mmol/L) and MnCl_2_ (2 mmol/L). Steady-state current amplitudes were calculated at the end of a 3 s step to −125 mV (holding potential, −35 mV). A two-step protocol was used to assess the voltage dependence of the current, with a first step to a test voltage in the range −35/−135 mV and a second step to −120 mV. Test step durations varied from 10 s at −35 mV to 5/7.5 s at −135 mV to allow full current activation at each voltage. The Boltzmann distribution (*y* = 1/(1+exp((*V*−*V*_1/2_)/s): *V*, voltage; *y*, fractional activation; *V*_1/2_, half-activation voltage; *s*, inverse-slope factor) was used to fit experimental data-points. Fully activated *I*_f_ current–voltage (*I*/*V*) relationships were obtained according to a previously published protocol^57^. Shifts of the *I*_f_ activation curve induced by isoproterenol (30 nM) or acetylcholine (30 nM) were measured near the midpoint of the activation curve (−75 mV) as previously reported^58^.

### Local Ca^2+^ releases in permeabilized SA node cells

SA node pacemaker cells were isolated from SA node tissue of 3-month-old mice and permeabilized using 0.01% saponin. After saponin washout, the solution was changed to a recording solution that contained the following (in mmol/L): fluo-4 pentapotassium salt, 0.03; CaCl_2_, 0.099 (free [Ca^2+^] ∼ 50 nM); C_4_H_6_NO_4_K (dl-aspartic acid potassium salt), 100; KCl, 25; NaCl, 10; MgATP, 3; MgCl_2_, 0.81 (~1 mM free Mg^2+^); Hepes, 20; EGTA, 0.5; phosphocreatine, 10; creatine phosphokinase (5 U/mL); pH 7.2. The cytosolic free Ca^2+^ at given total Ca^2+^, Mg^2+^, ATP, and EGTA concentrations was calculated using a computer program (WinMAXC 2.50, Stanford University). Spontaneous characteristics of Local Ca^2+^ Releases (LCR) were measured by confocal microscopy in fixed free [Ca^2+^] as previously described^59^. The amplitude of individual LCRs was expressed as peak value (*F*) normalized to minimal fluorescence (*F*_0_). LCR spatial size (FWHM) was indexed as the full width at half-maximum amplitude. LCR duration (FDHM) was characterized as the full duration at half-maximum amplitude. The number of LCRs was normalized per 100 µm of the line-scan image and during a 1 s time interval. The Ca^2+^ signal of an individual LCR was estimated as previously described^59^. *M* = FWHM × FDHM × (ΔCa^2+^, nmol/L)/2. The Ca^2+^ signal of the LCR ensemble was estimated by integrating the Ca^2+^ signal produced by each LCR and normalized per 100 µm of the line-scan image and during a 1 s time interval^59^.

### SA cell immunohistochemistry

To localize the expression of HCN4 in situ, 5 µm paraffin-embedded SA node sections were stained with a monoclonal anti-HCN4 antibody (1:800, ab85023, Abcam) employing the Dako EnVision+ System-HRP (DAB) kit (#K4006, CA, USA). The 3,3′-diaminobenzidine (DAB) substrate-chromogen reaction (brown color) was visualized using a Leica microscope. High-resolution/high-magnification (×400) digital images of representative cross-sections of SA node bodies were acquired using a computerized imaging analysis system (Metamorph, University Imaging).

To visualize the expression of phospholamban (PLN), freshly isolated SA cells were fixed, blocked, and permeabilized using the Image-iT Fixation/Permeabilisation kit (Life Technologies). Immunohistochemistry was undertaken using 1:200 primary mouse monoclonal anti-PLN (A010-14, Badrilla, Leeds, UK) and 1:1000 secondary Alexa Fluor 488 donkey anti-mouse (A212202, Life Technologies) antibodies. Nuclear counterstaining was with 4′,6-diamidino-2-phenylindole (DAPI). Fluorescence images were visualized using a Zeiss LSM710 confocal scanning microscope with a 63 × 1.4 NA immersion oil objective and images recorded with ZEN 2 acquisition software (Zeiss, Germany).

### iSAB and aCaB generation

Generation of iSABs (induced sinoatrial bodies) and aCaBs (antibiotic-selected cardiac bodies) was performed as previously described^27,28^. In brief, murine cell lines^27^ were used to generate iSABs and aCaBs and grown in high-glucose DMEM with stable glutamine (GIBCO) containing the following: 10% FBS Superior (Biochrom), 100 µM non-essential amino acids (GIBCO), 1% penicillin/streptomycin (GIBCO), and 100 μM β-mercaptoethanol (Sigma) in the presence of 1,000 U/mL of leukemia inhibitory factor (LIF, Millipore). Differentiation was performed by hanging drop culture for 2 days using 1,000 cells as starting material for one EB (embryoid body) in Iscove’s basal medium (Biochrom) containing the following: 10% FBS (Biochrom), 100 µM non-essential amino acids (GIBCO), 1% penicillin/streptomycin (GIBCO), and 450 µM 1-thioglycerol. Cells were differentiated for an additional 4 days in suspension culture, and at day 6 of differentiation 15 EBs were seeded onto one well of a 24-well-plate. At day 8 post-seeding, antibiotic selection was initiated using 400 µg/mL G418 (Biochrom). Four days thereafter, aCaBs and iSABs were isolated by treatment with 6,000 U/mL Collagenase IV (GIBCO) for 30 min. Single cells were obtained by further dissociation of the bodies using 100% Accutase (Affymetrix) for 15 min. Potential mycoplasma contamination was routinely controlled for twice a week using the PCR based MycoSPY kit system (Biontex). The iSABs were generated according to Rimmbach et al.^28^

ELISA assessment of α AMPK Thr172 phosphorylation was performed using the PathScan Phospho-AMPKα (Thr172) Sandwich ELISA Kit (Cell Signaling Technology Inc., USA) according to the manufacturer’s protocol. For this, 60 µg/mL protein was isolated from iSABs at 0, 15 and 60 min following treatment with 100 µM Compound 991, 0.5 mM AICAR or control and subjected to ELISA. Experiments were performed with four biological replicates, each of which was analyzed using two technical replicates.

### iSAB RNA sequencing and data analysis

For library generation and sequencing, cultured adherent cells were drained from the culture medium, washed, and directly lysed by addition of lysis buffer^60^. A total of 1 µL of this lysate was used for cDNA synthesis and amplification with the SMARTer kit (Clontech, Mountain View CA, USA) according to the manufacturer’s instructions. In brief, cDNA synthesis was initiated by annealing a polyA-specific primer and adding a reverse transcriptase with terminal transferase activity. The newly synthesized first strand cDNA was then tailed first with a homopolymer stretch by terminal transferase and then with a specific amplification tag by template switching. The resulting double-tagged cDNA was amplified by PCR, fragmented by sonication (Bioruptor, Diagenode, Liege, Belgium; 25 cycles 30 s on/30 s off) and converted to barcoded Illumina sequencing libraries using the NEBnext Ultra DNA library preparation kit (New England BioLabs, MA, USA). After PCR enrichment the libraries were purified with AmpureXP magnetic beads (Beckman-Coulter, CA, USA) and quantified on a Bioanalyzer 2100 (Agilent, CA, USA). Libraries were pooled at equimolar amounts and sequenced on an Illumina GenomeAnalyzer IIx in single-read mode with a read-length of 78 nucleotides and a depth of 21–32 million raw reads per replicate.

We performed adapter clipping and quality trimming procedures for data pre-processing^61^. We aligned the reads to the mm9 genome with the aid of TopHat2^62^. Differential expression analysis was performed using Cufflinks2/Cuffdiff2^63,64^. We considered genes with >2-fold change and a *q* value < 0.05 as significantly differentially expressed. The gene annotation, including functional annotation clustering and functional classification, was performed with DAVID and based on gene ontology terms^65^. We used our openly available RNA sequencing pipeline (TRAPLINE) for data analysis^66^.

### WGCN analysis

Weighted gene coexpression network analysis was performed by applying the R package “WGCNA” to RNASeq data^67^. We first constructed the topological overlap matrix (TOM) of all investigated transcripts (~30,000) using the soft thresholding method. We calculated the eigenvalues of the transcripts and evaluated adjacency based on distance. We subjected transcripts to hierarchical clustering (average linkage) and assigned transcripts with the dynamic hybrid method into groups. We computed connectivity based on the interaction partners (*k*) and evaluated gene significance, representing module membership. Finally, we computed a network screening analysis using the WGCNA package to distinguish between true positive results and noise.

### Gene ontology analysis

Networks were built using several applications in Cytoscape^68^. ClueGo was used to visualize and cluster the gene annotation terms into groups^69^. The KEGG and Panther pathway databases were used to obtain specific gene annotations^70–72^. The network interaction graph was built with the aid of enhanced Graphics and integrates fold change values (http://apps.cytoscape.org/apps/enhancedGraphics). Interactions between mRNAs were identified with “Agilent literature search” and are based on validated publications (http://apps.cytoscape.org/apps/agilentliteraturesearch).

### Isolation and culture of primary murine SA cells

SA cells were isolated as previously described^31^ from 2–4-month-old male C57BL/6J (Jackson Laboratories) or homozygous R299Q γ2 mice. Mice were anesthetized by inhalation of isoflurane prior to killing. Hearts were rapidly excised, the ventricles and left atria removed, and the SA node dissected from the remaining right atrial tissue at 35 °C in a heparinized Tyrode’s solution, which consisted of the following (in mM): 140 KCl, 5.4 KCl, 1.2 KH_2_PO_4_, 5 HEPES, 5.55 d-Glucose, 1 MgCl_2_, 1.8 CaCl_2_, with a pH adjusted to 7.4 with NaOH. SA node tissue was enzymatically digested by 4.75 U elastase (Worthington Biochemical), and 3.75 mg Liberase TM (Roche) for 15 min at 35 °C in a modified Tyrode’s solution, containing (in mM) 140 NaCl, 5.4 KCl, 1.2 KH_2_PO_4_, 5 HEPES, 18.5 d-glucose, 0.066 CaCl_2_, 50 taurine, 1 mg/mL bovine serum albumin (BSA), with pH adjusted to 6.9 with NaOH. Following digestion, tissue was transferred to a modified KB solution (in mM: 100 potassium glutamate, 10 potassium aspartate, 25 KCl, 10 KH_2_PO_4_, 2 MgSO_4_, 20 taurine, 5 creatine, 0.5 EGTA, 20 glucose, 5 HEPES, and 0.1% BSA; pH adjusted to 7.2 with KOH) at 35 °C and SA cells dissociated by mechanical trituration with a fire-polished glass pipette. The calcium concentration of the cell suspension was gradually increased to 1.8 mM.

Following calcium re-adaptation, SA cells were pelleted (at ~3000 RPM) and the supernatant carefully aspirated. For plating, SA cells were suspended in plating media, which contained Media199 (#M4530, Sigma) supplemented with 10 mM 2,3-butanedione monoxime (BDM), 10,000 U penicillin/10 mg streptomycin, and 5% (v/v) FBS. Glass coverslips were prepared by coating 12-mm diameter coverslips for ~1 h at 37 °C with 100 ng/mL mouse laminin (BD Biosciences, San Jose, CA, USA) diluted in phosphate-buffered saline (PBS). Excess laminin/Tyrode’s was removed immediately before cell plating. SA cells were plated such that the cells from one mouse SA node were seeded onto one 12-mm round coverslip. SA cells were allowed to settle and adhere to the coverslip for 4–6 h at 37 °C in an atmosphere of 95% air/5% CO_2_ before changing the media to culture media, which consisted of Media199 supplemented with 0.1 mg/mL bovine serum albumin (BSA; Sigma), 10 mM BDM, 10 μg/mL insulin, 5.5 μg/mL transferrin, 5 ng/mL selenium (ITS; Sigma), and 10,000 U penicillin/10 mg streptomycin. Culture media was exchanged every 24 h.

### Adenoviral transduction of primary murine SA cell cultures

SA cells on each coverslip were counted immediately prior to viral delivery. Adenoviral infections were performed on the same day as isolation in serum-free culture media at a multiplicity of infection (MOI) of 100 (100 infectious agents per target cell) as described^31^. Adenoviruses Ad-mCherry, Ad-mCherry-mPrkag2 (WT)-FLAG (i.e. Ad-WT γ2) and Ad-mCherry-mPrkag2(R299Q)-FLAG (i.e. Ad-R299Q γ2) were constructed, amplified and purified by Vector BioLabs (Philadelphia, PA, USA). SA cells were incubated with virus-containing media overnight (~12–14 h) and replaced with fresh culture media the following morning.

A fragment of a coverslip bearing SA cells was transferred to the recording chamber of an inverted microscope. During all experiments, cells were constantly perfused (1–2 mL/min) with extracellular solution at 35 ± 1 °C. SA cells were identified by their characteristic morphology, small size, and generation of spontaneous action potentials. Patch clamp recordings used borosilicate glass pipettes with resistances of 1.5–3 MΩ. Data were acquired at 5–20 kHz and low-pass filtered at 1 kHz using an Axopatch 1D or 200B amplifier, Digidata 1322a or 1440a A/D converter and ClampEx software (Molecular Devices). The fast component of pipette capacitance was minimized in all recordings using the patch-clamp amplifier. Membrane capacitance was estimated from responses to 10 mV test pulses using the membrane test function in ClampEx. Spontaneous beating rates were recorded from SA cells in the whole-cell configuration in current-clamp mode without injected current. Cells were constantly perfused with normal Tyrode’s solution (in mM: 140 NaCl, 5.4 KCl, 1.2 KH_2_PO_4_, 5 HEPES, 5.55 glucose, 1 MgCl_2_, 1.8 CaCl_2_; pH adjusted to 7.4 with NaOH). The intracellular (pipette) solution was composed of the following (in mM): 140 K-Aspartate, 10 HEPES, 1.8 MgCl_2_, 10 NaCl, 0.1 EGTA, 0.02 CaCl_2_; pH adjusted to 7.2 with KOH. Recordings were only made from infected cells, identified by mCherry fluorescence, in each culture. Viral transduction efficiency was essentially 100%, with no difference in efficiency between the different constructs and with each cell in the dataset infected. Beating rates were determined from averages of the instantaneous frequency during 15–30 s recording windows in the presence of 1 nM Isoproterenol (ISO; Calbiochem) in the bath.

### Primary rabbit SA cell culture and adenoviral transduction

Adult rabbits were treated in accordance with the NIH Guide for the Care and Use of Laboratory Animals (animal protocol number: 034LCS2016). Single, spindle-shaped, spontaneously beating SA cells were isolated from the hearts of New Zealand rabbits (Charles River Laboratories, Wilmington, MA, USA) as described previously^73^. To generate cultured SA cells (c-SANC), freshly isolated SA cells were diluted 20 times with serum-containing medium, and centrifuged for 10 min at 500 rpm. Following aspiration of the supernatant, cells were plated at a density of 0.5×10^4^ per cm^2^ on laminin pre-coated (20 µg/mL, Sigma-Aldrich) glass-bottom dishes for culture. The serum-containing medium^74^ contained a 73% salt solution (in mmol/L: NaCl 116, KCl 5.4, MgCl_2_ 0.8, NaH_2_PO_4_ 0.9, d-Glucose 5.6, Hepes 20, CaCl_2_ 1.8, NaHCO_3_ 26), 20% M199 (Sigma-Aldrich), in the presence of (in mmol/L) creatine 5, taurine 5, insulin–transferrin–selenium-X 0.1%, 4% fetal bovine serum, 2% horse serum, and 1% penicillin and streptomycin (pH = 7.4 at 37 °C).

Cells were incubated in serum-containing medium for the first 24 h, and then cultured in serum-free medium for adenoviral infection. Adenoviruses Ad-mCherry-mPrkag2 (WT)-FLAG and Ad-mCherry-mPrkag2(R299Q)-FLAG were introduced into c-SANC by an acute adenoviral gene-transfer technique using a MOI of 1,000 for 48 h. In addition to no adenoviral culture control, Ad-mCherry was employed as an adenoviral vector control. All functional and immune-labeling experiments were performed with cells cultured for 72 h.

### Measurement of spontaneous beating rate and immuno-labeling of cultured rabbit SA cells post-adenoviral transduction

In the three adenoviral-treated groups, the density of mCherry was employed as a guide to successful infection visualized via laser 543 nm (1 or 1.5% laser power). The bath superfusion solution contained (in mmol/L): NaCl 140, KCl 5.4, MgCl_2_ 1, HEPES 5, CaCl_2_ 1.8, and Glucose 5.5 (pH = 7.4). All functional measurements were performed at 34 ± 0.5 °C. Spontaneous contraction was recorded via the line-scan of transmission image using a confocal microscope (Zeiss LSM510, Carl Zeiss Inc., Germany) and the spontaneous beating rate calculated from the average duration between peak onsets^74^.

After each functional measurement, c-SANC were fixed with 4% Paraformaldehyde (10 min) for immuno-labeling. After permeabilization (1% Triton X-100 in PBS, 15 min) and blocking (PBS with 2% IgG-free BSA, 5% donkey serum, 0.02% NaN_3_, and 0.1% Triton X-100, 4 h), c-SANC were incubated with primary anti-Flag M2 (1:100, Sigma) at 4 °C overnight and then stained with Cy5-conjugated donkey anti-mouse secondary antibody (1:1000, Jackson ImmunoResearch laboratories, USA) for 1 h^74^. In the negative control group, only secondary antibody was applied. A 633 nm or 543 nm (at 10% power) laser was employed to excite the fluorophore Cy5 or mCherry via a confocal microscope (Zeiss LSM510, Carl Zeiss Inc., Germany). For semi-quantification, the average density of Flag and mCherry were measured using ImageJ (1.48v, National Institute of Health, USA), with the nuclear area excluded.

### Echocardiography

Transthoracic echocardiography was undertaken as previously described^47^. In brief, Sox2cre γ2 AMPK knockout and WT Cre+ mice aged ~2 months underwent general anesthesia with isoflurane (3–4% induction and 1–1.5% maintenance) in oxygen administered via nosecone. Images were acquired on a heated platform using a Vevo 2100 Imaging System (VisualSonics, Toronto) and analyzed off-line blind to genotype using Vevo software.

### Surface ECG recordings

Anesthetized ECG recordings were obtained under light isoflurane (1.25%) anesthesia. Four fine needle electrodes were positioned subcutaneously and a 5 min period of equilibration undertaken, prior to a 10 min ECG recording with filtering and amplification of signals by Bio Amplifier (ADInstruments, Oxford, UK). ECG waveforms were then analyzed off-line using LabChart software (ADInstruments) blind to genotype.

### Voluntary wheel running and intact SA node/atrial preparation rate measurement

Sox2cre γ2 AMPK knockout (Homo fl Cre+) and WT Cre+ mice aged 3 months were singly housed in cages containing a freely rotating, angled running track (Lillico, UK), with wheel rotations monitored by use of a reed switch connected to a computerized exercise monitoring system (Micro 1401, CED, Cambridge) as described^75^. Mice were allowed to acclimatize to single housing for several days and then data was continuously recorded for 10 weeks. During this period, cage disturbance was kept to a minimum, with ad libitum access to the running wheel. Data acquisition and analysis blind to genotype were carried out using Spike2 software (CED). For determination of intrinsic rate as part of these experiments, atria were rapidly dissected and transferred to a 2 mL organ bath containing Tyrode solution at 37 ± 0.5 °C, where they were allowed to equilibrate as previously described^37^.

### Statistical analysis

Data are presented as means ± s.e.m. Numbers of mice were determined by power calculations using in-house and available published data. Unless otherwise stated, data were analyzed with Student’s *t*-test or one-way analysis of variance (ANOVA) followed by Holm–Sidak’s multiple comparisons test. Non-parametric data were analyzed by Kruskal–Wallis test followed by Dunn’s multiple comparisons test. Statistical analysis was performed with GraphPad Prism Software (v6.0, CA, USA) with *P* < 0.05 considered statistically significant.

### Data availability

Microarray and RNASeq datasets generated for this study have been deposited in the Gene Expression Omnibus (Accession Number GSE73047) and the Sequence Read Archive (Accession Number SRS1064711), respectively.

## Electronic supplementary material

Supplementary Information

Description of Additional Supplementary Files

Supplementary Movie 1

Supplementary Movie 2
