# Supplementary material for: Mammalian γ2 AMPK regulates intrinsic heart rate
Source: Nat Commun. 2017 Nov 2;8:1258. doi: 10.1038/s41467-017-01342-5 (PMC5668267; doi:10.1038/s41467-017-01342-5)
Supplement: Supplementary file 1 — Supplementary Information [file 41467_2017_1342_MOESM1_ESM.pdf]

SUPPLEMENTARY FIGURES AND LEGENDS

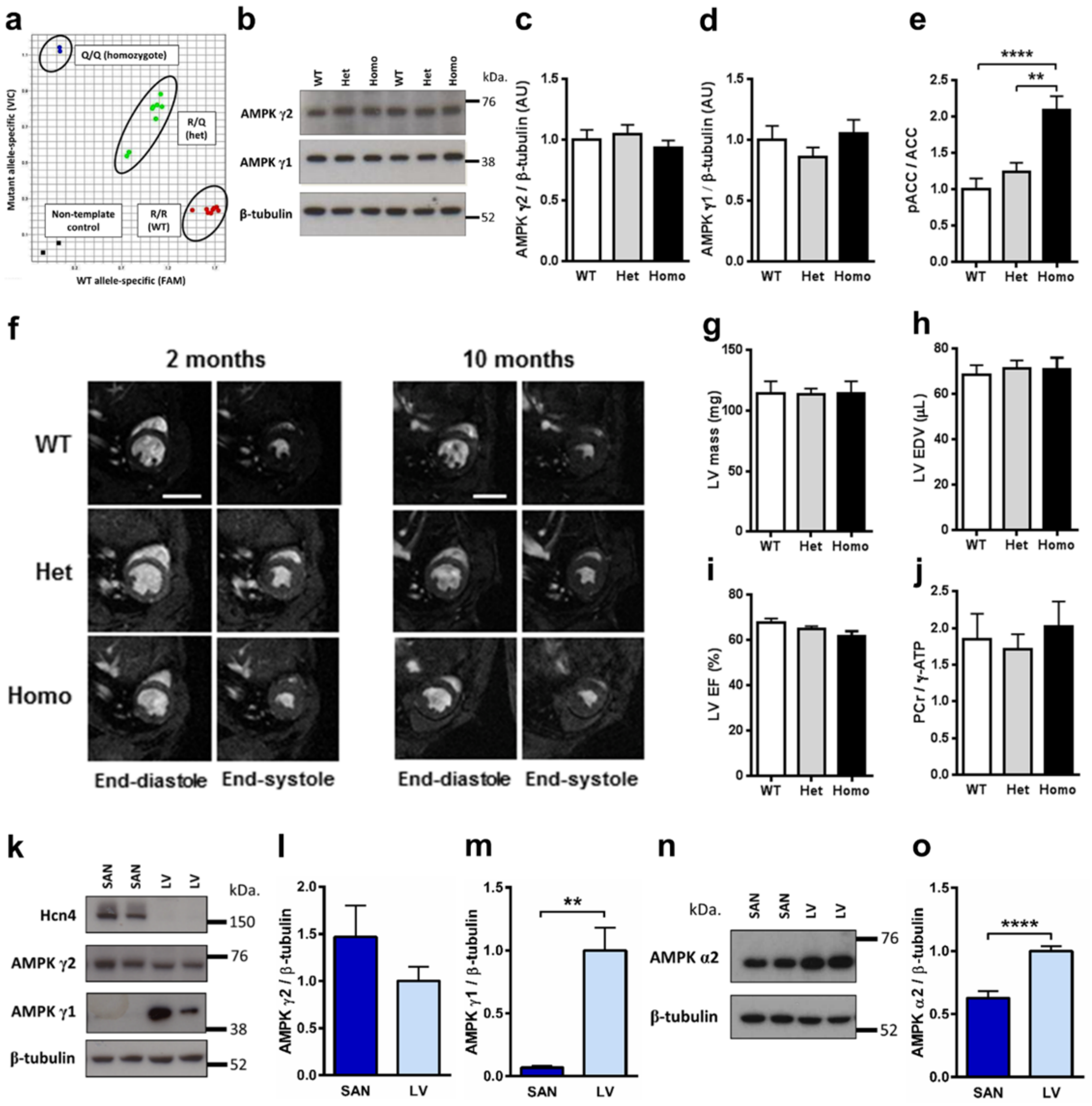

Supplementary Figure 1. Validation and cardiac phenotype of the R299Q γ2 AMPK knock-in mouse model. (**a**) Allelic discrimination plot obtained using WT γ2- and R299Q γ2-specific probes on whole heart cDNA. (**b**) Representative western blot of whole heart tissue for γ AMPK isoforms from R299Q γ2 (Het, heterozygous; Homo, homozygous) mice and wild-type (WT) controls, with β-tubulin as loading control. (**c, d**) Densitometry analysis of western blot of whole heart tissue from R299Q γ2 and WT mice for (**c**) γ2 and (**d**) γ1 AMPK isoforms. Relative protein levels are presented normalised to β-tubulin (n = 11-15). (**e**) Densitometry analysis of western blots of whole heart tissue from R299Q γ2 and WT mice for phospho-acetyl-CoA Carboxylase (pACC) (n = 11-15). (**f**) Representative mid-ventricular, end-diastolic and end-systolic short-axis cine-MRI images at 2 and 10 months of age (scale bar 5 mm). (**g-i**) Cine MRI analysis of left ventricular (LV) mass (**g**), end-diastolic volume (EDV) (**h**) and ejection fraction (EF) (**i**) in R299Q γ2 and WT mice aged 10 months (n = 8-19). (**j**) *In vivo* myocardial PCr/γ-ATP ratio determined by ^{31}P MRS (Magnetic Resonance Spectroscopy) (n = 7-19). (**k-m**) Western blot (**k**) and densitometry (**l, m**) of γ2 and γ1 AMPK in normal murine SA node and LV, together with SA node positive (HCN4) and loading (β-tubulin) controls (n = 6-8). (**n, o**) Western blot (**n**) and densitometry (**o**) of α2 AMPK in normal murine SA node and LV (n = 4-10). Uncropped western blots are shown in Supplementary Fig. 10. In **c-e**, **g-j,** one-way analysis of variance (ANOVA) followed by Holm-Sidak's multiple comparisons test was performed; in **l, m, o** Student's t-test was performed. **$P < 0.01$, ****$P < 0.0001$. In **c-e, g-j, l,m,o** data are shown as means ± s.e.m.

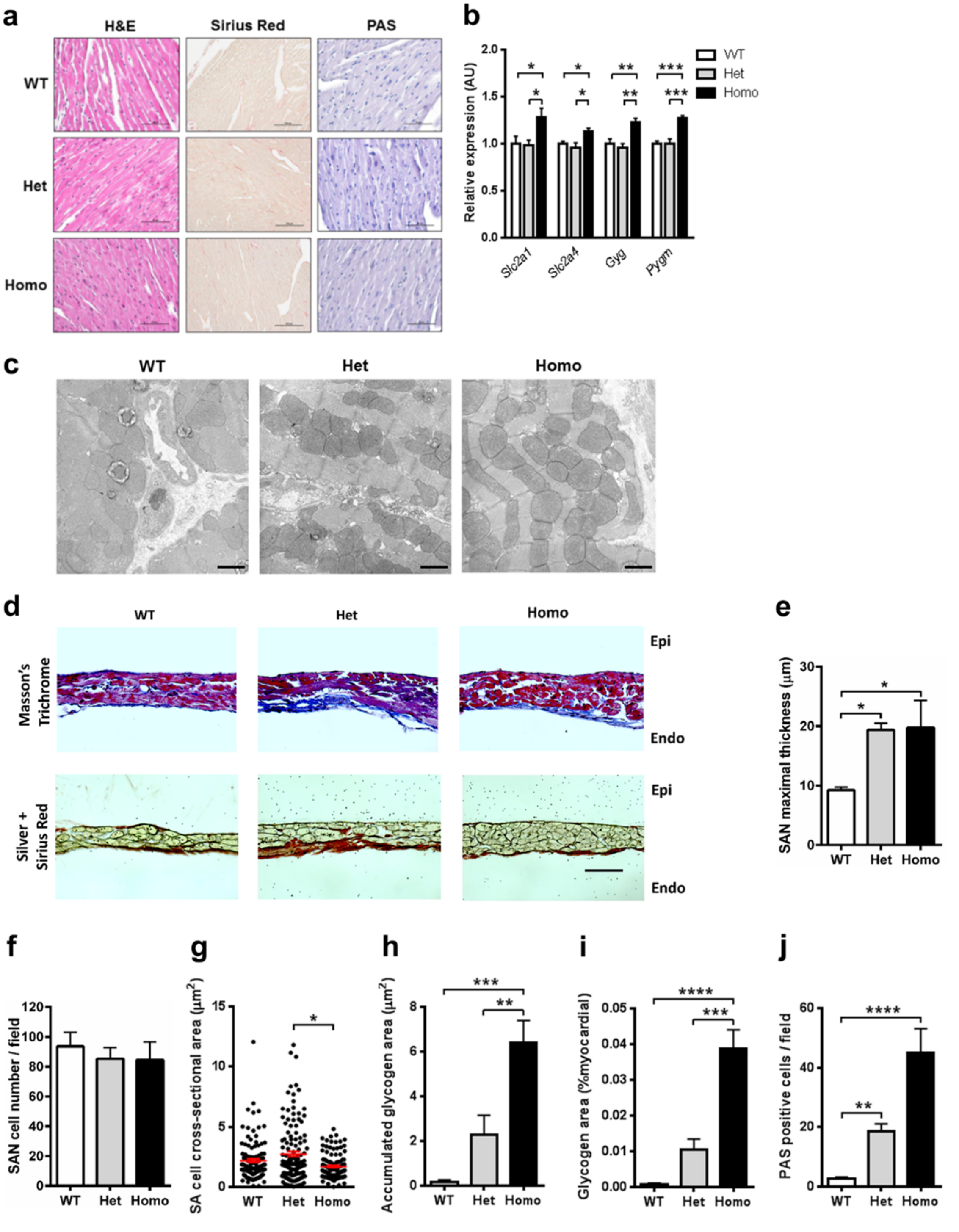
a
H&E
Sirius Red
PAS
WT
Het
Homo
b
Relative expression (AU)
2.0
1.5
1.0
0.5
0.0
WT
Het
Homo
Slc2a1
Slc2a4
Gyg
Pygm
c
WT
Het
Homo
d
WT
Het
Homo
Masson's Trichrome
Silver + Sirius Red
Epi
Endo
Epi
Endo
e
SAN maximal thickness (μm)
30
20
10
0
WT
Het
Homo
f
SAN cell number / field
120
100
80
60
40
20
0
WT
Het
Homo
g
SA cell cross-sectional area (μm2)
15
10
5
0
WT
Het
Homo
h
Accumulated glycogen area (μm2)
8
6
4
2
0
WT
Het
Homo
i
Glycogen area (%myocardial)
0.05
0.04
0.03
0.02
0.01
0.00
WT
Het
Homo
j
PAS positive cells / field
60
40
20
0
WT
Het
Homo

Supplementary Figure 2. Cardiac histology and glycogen content of R299Q γ2 AMPK knock-in mice. (**a**) Histological appearances of hearts from R299Q γ2 and WT mice aged 12 months stained for haematoxylin and eosin (H&E, scale bar 50 µm), sirius red (scale bar 100 µm) and periodic acid-Schiff (PAS, scale bar 50 µm). (**b**) Cardiac relative expression of genes (by qRT-PCR) involved in glucose uptake and glycogen metabolism from R299Q γ2 and WT mice (n = 7-8). (**c**) Cardiac ultrastructure of R299Q γ2 and WT mice aged 12 months by transmission electron microscopy (scale bar 1 µm). (**d**) Representative SA node sections stained with Masson's trichrome (upper row) or silver staining with sirius red (lower row) (scale bar 10 µm). (**e, f**) Maximal SA node thickness (**e**) and SA node cell number per field (**f**) in R299Q γ2 and WT mice. (**g**) Cross-sectional area of individual SA cells from R299Q γ2 and WT mice. (**h-j**) Quantification of glycogen content from PAS-stained SA node sections of R299Q γ2 and WT mice (n = 12). In **b, e, f, h-j,** one-way analysis of variance (ANOVA) followed by Holm-Sidak's multiple comparisons test was performed; in **g,** the Kruskall-Wallis test followed by Dunn's multiple comparisons test was performed. *$P < 0.05$, **$P < 0.01$, ***$P < 0.001$, ****$P < 0.0001$. In **b, e-j,** data are shown as means ± s.e.m.

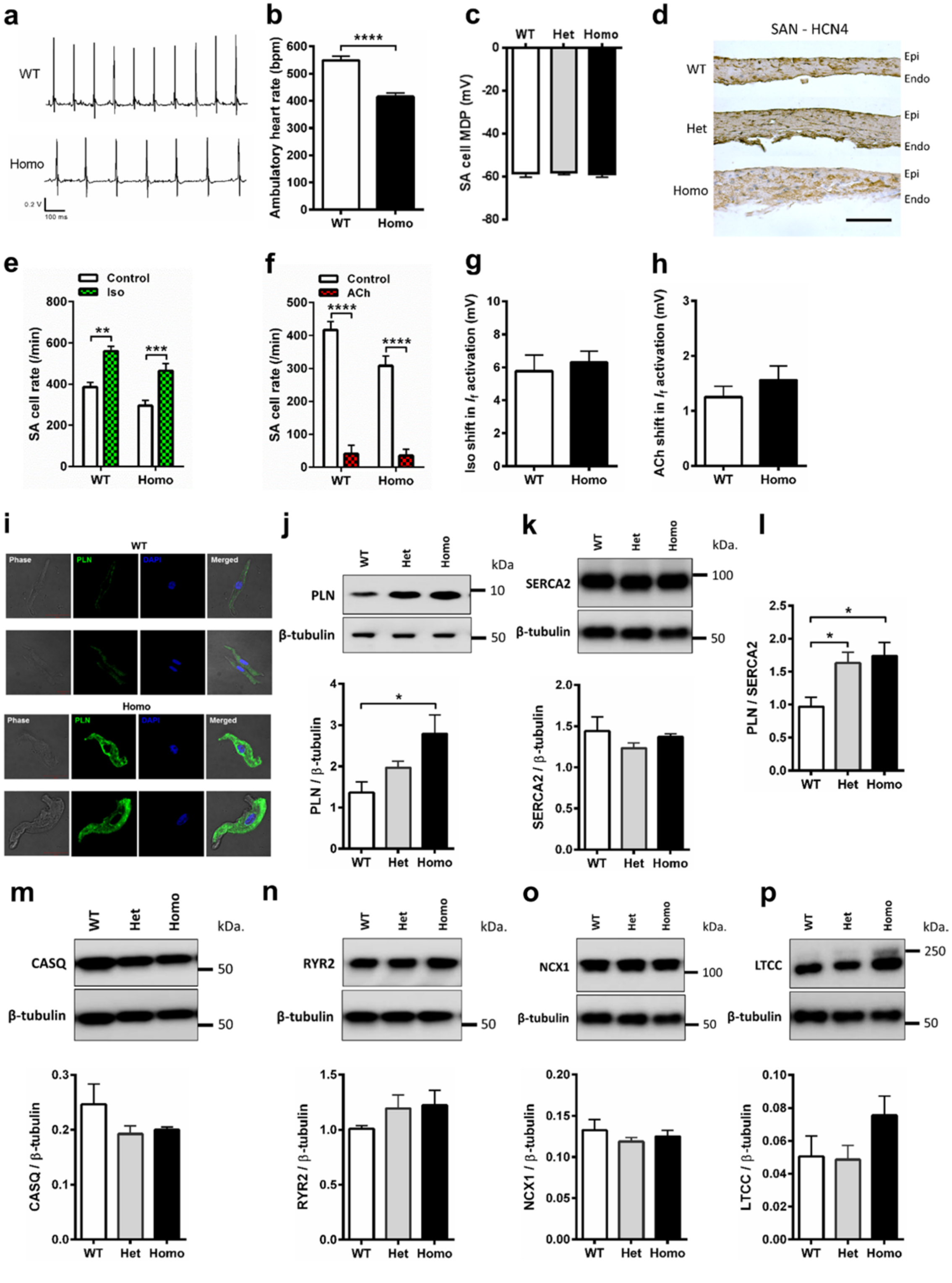
a
WT
Homo
0.2 V
100 ms
b
****
Ambulatory heart rate (bpm)
WT
Homo
c
WT Het Homo
SA cell MDP (mV)
d
SAN - HCN4
WT
Het
Homo
Epi
Endo
e
Control
Iso
**
***
SA cell rate (/min)
WT
Homo
f
Control
ACh
****
SA cell rate (/min)
g
Iso shift in I_f activation (mV)
h
ACh shift in I_f activation (mV)
i
WT
Phase
PLN
DAPI
Merged
Homo
j
WT
Het
Homo
kDa
PLN
β-tubulin
10
50
*
PLN / β-tubulin
k
SERCA2
β-tubulin
kDa.
100
SERCA2 / β-tubulin
l
PLN / SERCA2
m
CASQ
CASQ / β-tubulin
n
RYR2
RYR2 / β-tubulin
o
NCX1
NCX1 / β-tubulin
p
LTCC
250
LTCC / β-tubulin

Supplementary Figure 3. SA cell electrophysiology, HCN4 and Ca^{2+} clock component expression in R299Q γ2 mice. (**a, b**) Representative ambulatory telemetry-derived ECG recordings (**a**) and mean ambulatory HR (**b**) of WT and homozygous R299Q γ2 mice (*n* = 8 per genotype). (**c**) Maximum diastolic potential (MDP) of isolated SA cells from R299Q γ2 and WT mice (*n* = 5-11). (**d**) SA node sections stained with anti-HCN4 (scale bar 5 µm). (**e, f**) Mean SA cell spontaneous rate under control conditions or in response to stimulation with 30 nM isoproterenol (Iso, **e**) or 30 nM acetylcholine (ACh, **f**) (*n* = 6-8). (**g, h**) Mean shifts of the I_f activation curve induced by 30 nM Iso (**g**) or 30 nM ACh (**h**). Iso and ACh shifts are depolarizing and hyperpolarizing, respectively (*n* = 5-8). (**i**) Immunohistochemistry for total phospholamban (PLN, green) in freshly isolated SA cells from WT and homozygous R299Q γ2 mice (scale bar 20 µm). Nuclei were counterstained with DAPI (blue). (**j-p**) Western blot (and accompanying densitometry) of isolated SA node from WT and R299Q γ2 mice for the calcium clock components: phospholamban (PLN, **j**), sarco(endo)plasmic reticulum Ca^{2+}-ATPase (SERCA, **k**), calsequestrin (CASQ, **m**), cardiac ryanodine receptor (RYR2, **n**), Na^+/Ca^{2+}exchanger (NCX1, **o**) and L-type Ca^{2+} channel (LTCC, **p**), together with the PLN/SERCA2 ratio (**l**). Uncropped western blots with location of molecular weight markers are shown in Supplementary Fig. 10. In **b, g, h,** the Student's *t*-test was performed; in **c, j-p,** one-way analysis of variance (ANOVA) was performed; in **e, f,** two-way ANOVA was performed. *$P < 0.05$, **$P < 0.01$,***$P < 0.001$, ****$P < 0.0001$. In **b, c, e-h, j-p,** data are shown as means ± s.e.m.

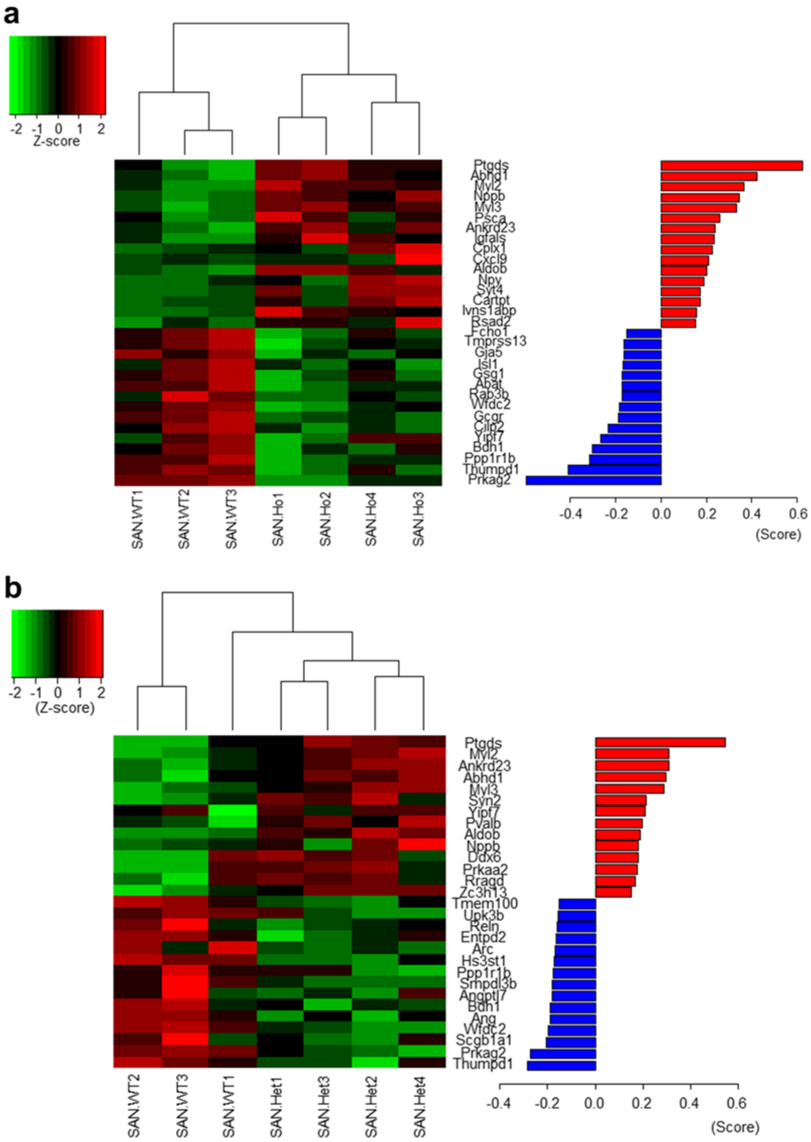

Supplementary Figure 4. Hierarchical cluster analysis of SA node microarray from R299Q γ2 mice. (**a, b**) Heat map plot of differentially expressed genes from SA node of (**a**) homozygous R299Q γ2 vs WT and (**b**) heterozygous R299Q γ2 vs WT mice. Genes were selected with the Kolmogorov-Smirnov test (score >0.15 or <-0.15). The Z-score is the standardized level of expression obtained by subtracting the mean from each value and dividing the results by the standard deviation. GSEA software was used to compute scores for each gene. Red and blue bars indicate up- and down-regulation, respectively.

Network 1 – Homo vs WT

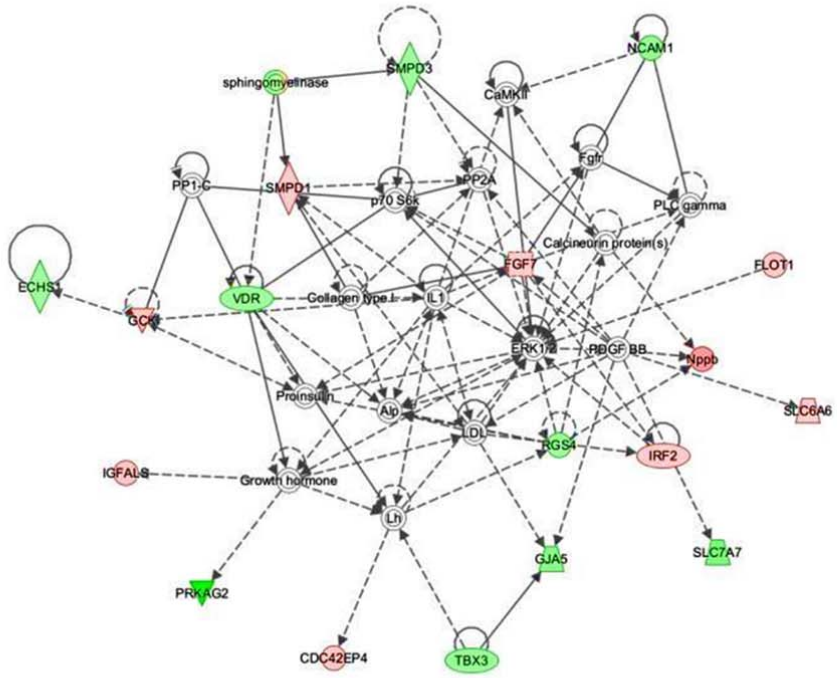

Network 2 – Homo vs WT

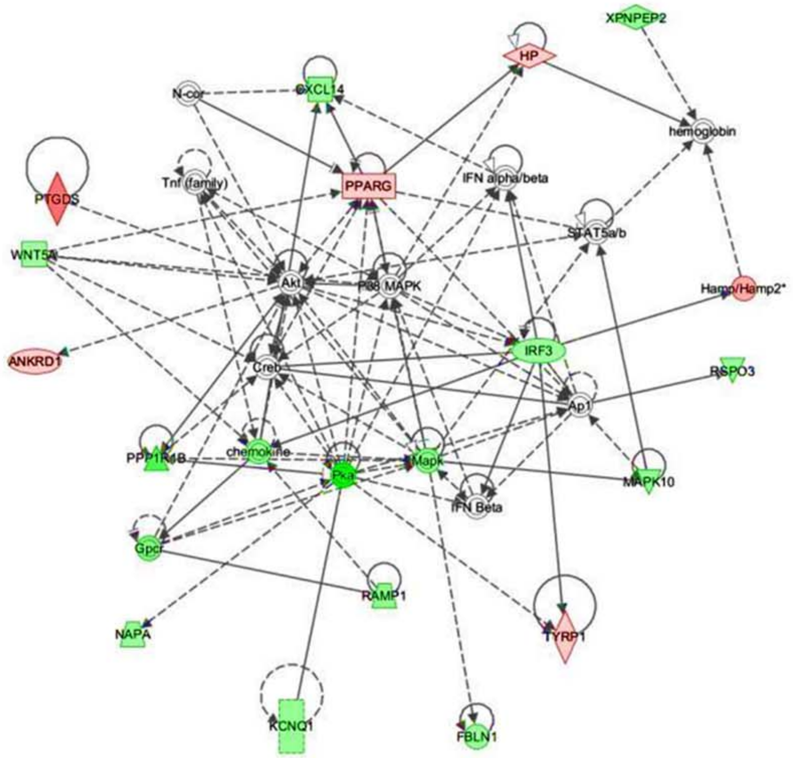

Network 3 – Homo vs WT

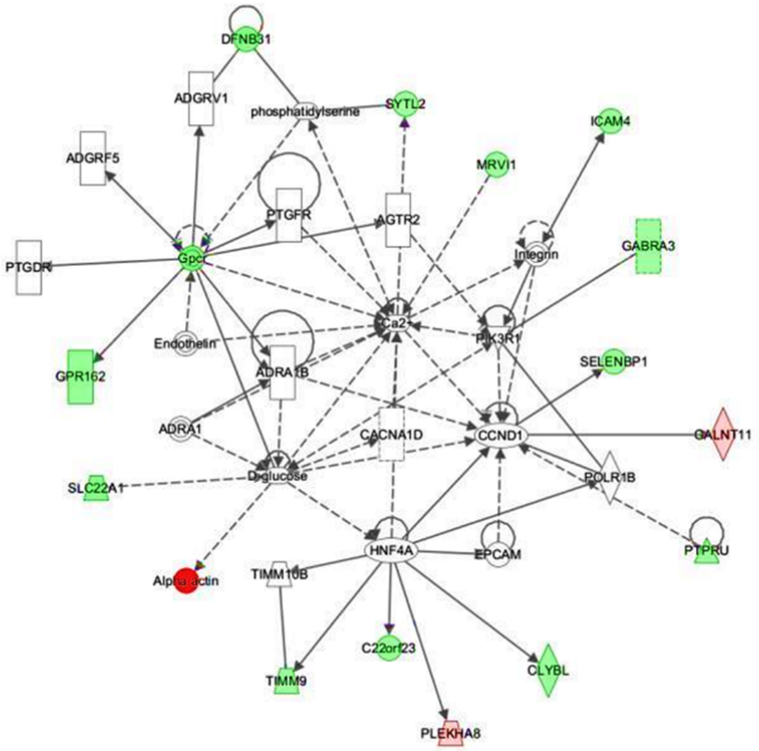

Network 4 – Homo vs WT

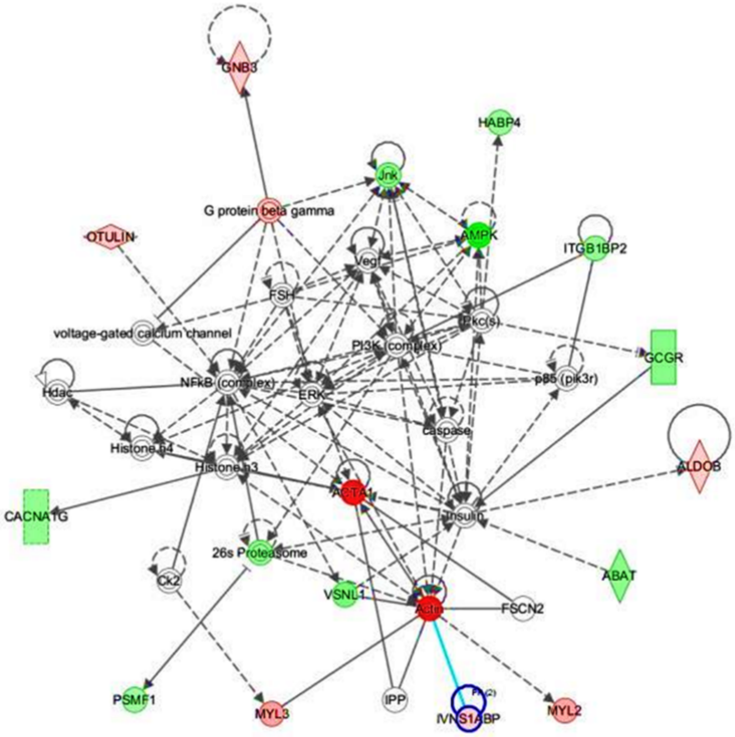

76

Supplementary Figure 5. Ingenuity pathway network analysis of homozygote R299Q γ2 versus WT differential SA node transcriptome. Red/green are genes up/down-regulated, respectively, in SA node of homozygous R299Q γ2 mice vs WT; dashed line - indirect interactions; solid lines - direct interactions. Detailed legend for symbols given in: http://ingenuity.force.com/ipa/articles/Feature_Description/Legend.

Network 1 – Het vs WT

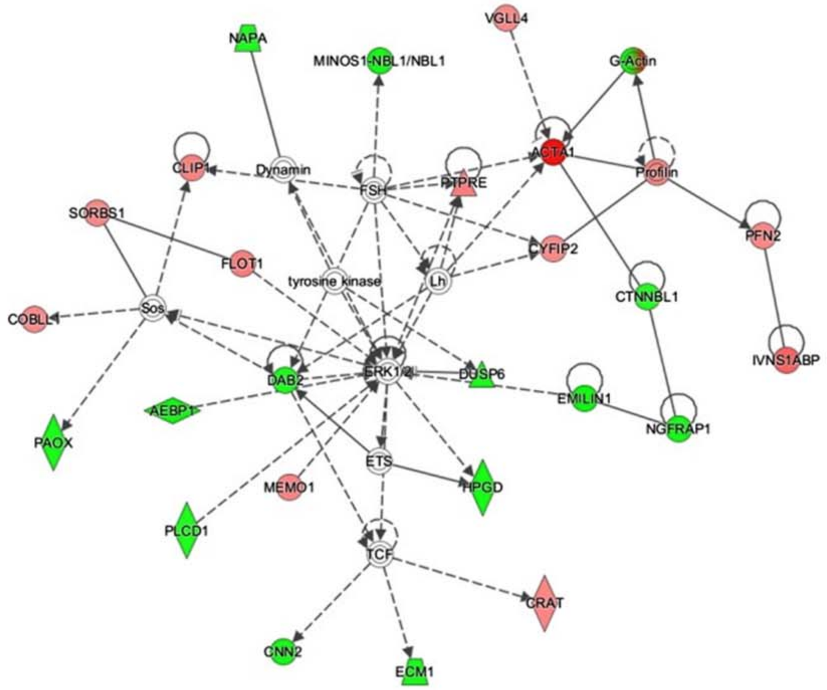

Network 2 – Het vs WT

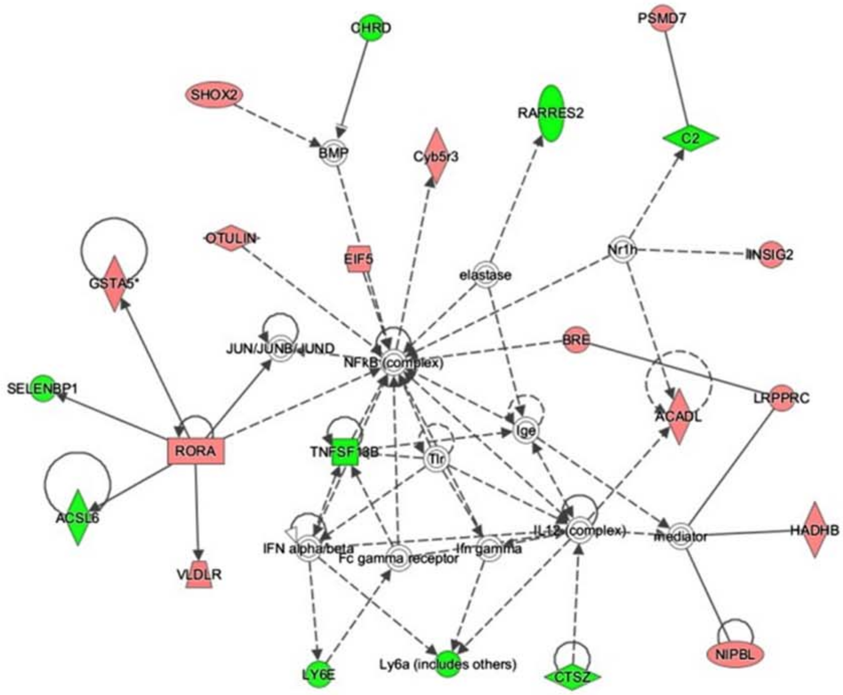

83

Network 3 – Het vs WT

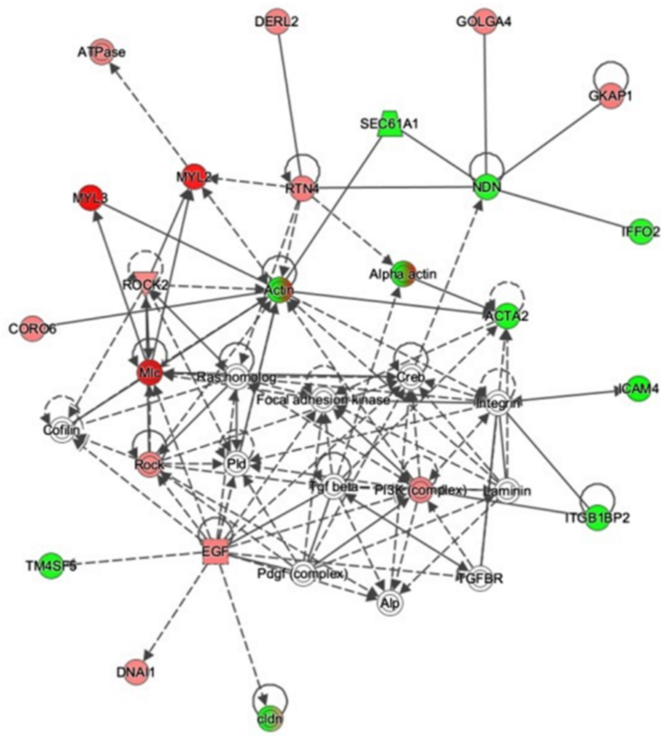

Network 4 – Het vs WT

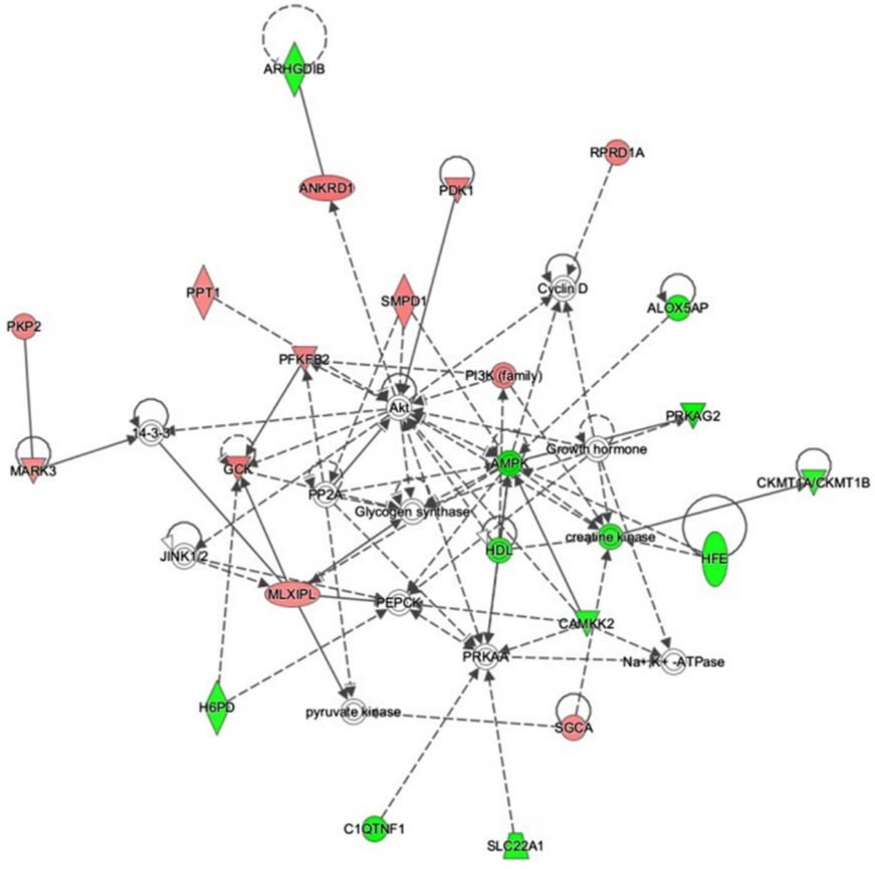

Network 5 – Het vs WT

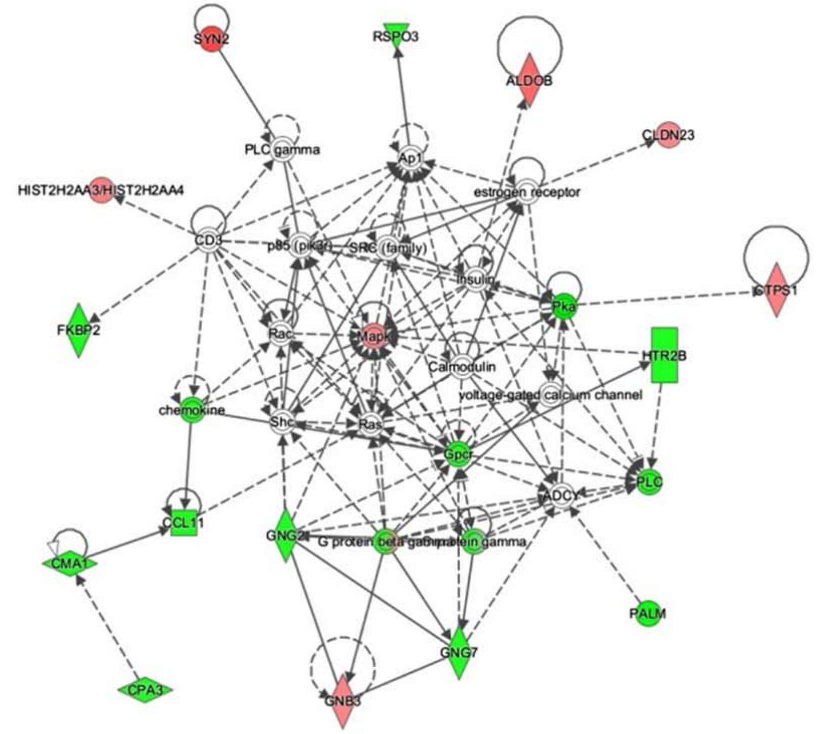

Network 6 – Het vs WT

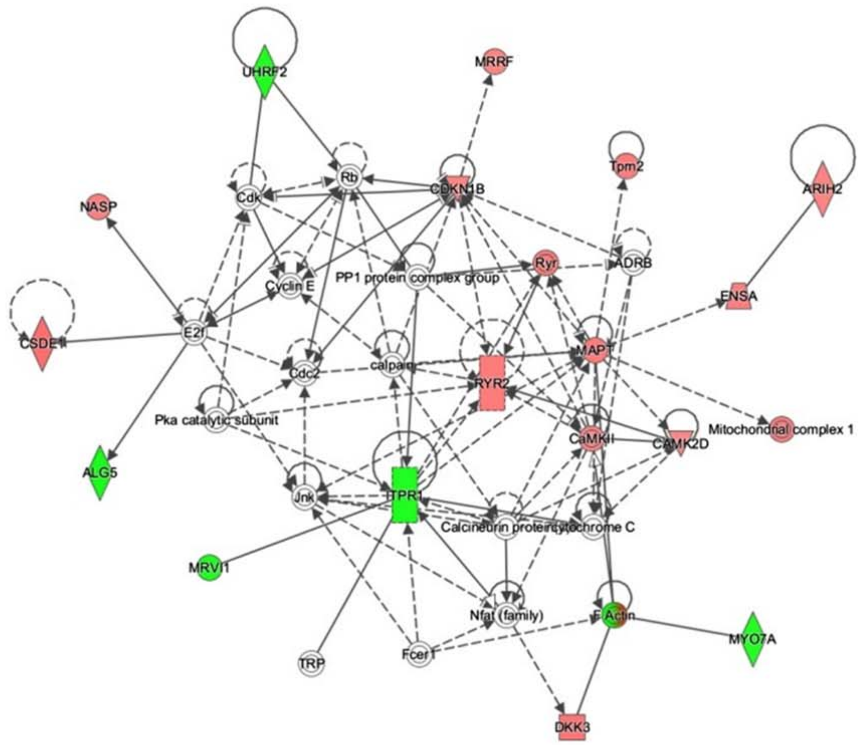

85

Supplementary Figure 6. Ingenuity pathway network analysis of heterozygote R299Q γ2 versus WT differential SA node transcriptome. Red/green are genes up/down-regulated, respectively, in SA node of heterozygous R299Q γ2 mice vs WT; dashed line - indirect interactions; solid lines - direct interactions. Detailed legend for symbols given in:

http://ingenuity.force.com/ipa/articles/Feature_Description/Legend.

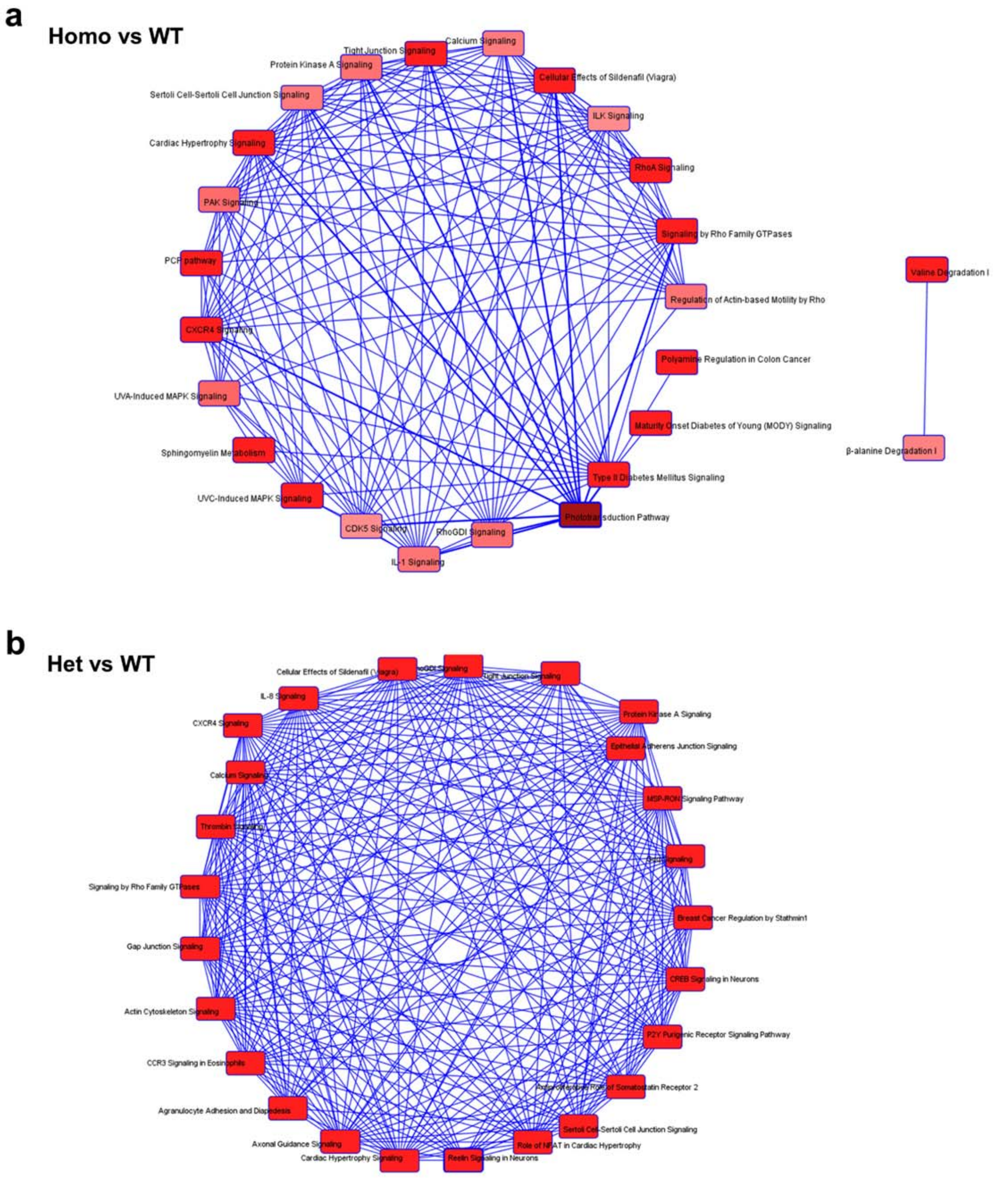

Supplementary Figure 7. Ingenuity pathway analysis identification of canonical pathways associated with R299Q γ2 AMPK differential SA node transcriptome. (**a, b**) Ingenuity pathway analysis identification of canonical pathways associated with R299Q γ2 AMPK differential SA node transcriptome. (**a**) Hmozygote R299Q γ2 versus WT SA node comparison. (**b**) Heterozygote R299Q γ2 versus WT SA node comparison.

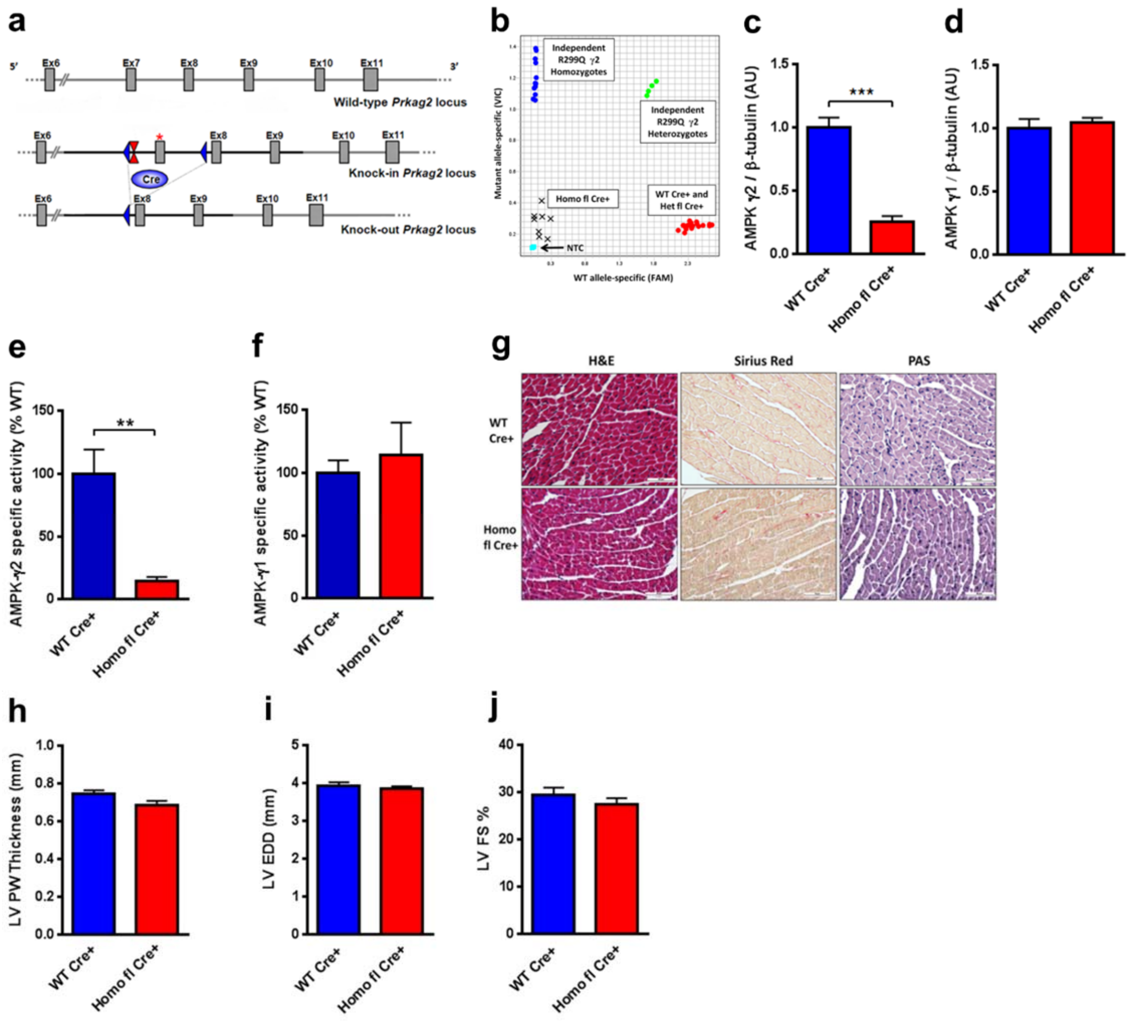
a
5'
Ex6
Ex7
Ex8
Ex9
Ex10
Ex11
3'
Wild-type Prkag2 locus
Knock-in Prkag2 locus
Cre
Knock-out Prkag2 locus
b
Independent R299Q γ2 Homozygotes
Independent R299Q γ2 Heterozygotes
Homo fl Cre+
WT Cre+ and Het fl Cre+
NTC
Mutant allele-specific (VIC)
WT allele-specific (FAM)
c
AMPK γ2 / β-tubulin (AU)
***
d
AMPK γ1 / β-tubulin (AU)
e
AMPK-γ2 specific activity (% WT)
**
f
AMPK-γ1 specific activity (% WT)
g
H&E
Sirius Red
PAS
WT Cre+
Homo fl Cre+
h
LV PW Thickness (mm)
i
LV EDD (mm)
j
LV FS %
WT Cre+
Homo fl Cre+

Supplementary Figure 8. γ2 AMPK knock-out mouse model validation and gross cardiac phenotype. (**a**) Illustration of strategy used to generate Sox2cre γ2 AMPK knock-out mice by Cre-mediated excision of R299Q mutated exon 7 of *Prkag2*. (**b**) Allelic discrimination plot illustrating absence of R299Q γ2–specific transcript in Sox2cre γ2 AMPK knock-out mice (Homo fl Cre+) and clustering with non-template controls (NTC). (**c, d**) Densitometry analysis of western blot of whole heart tissue from Homo fl Cre+ and Sox2cre+ control (WT Cre+) mice for γ2 (**c**) and γ1 (**d**) AMPK isoforms. Relative protein levels are presented normalised to β-tubulin (n = 4). (**e, f**) AMPK γ2- (**e**) and γ1- (**f**) specific activity of freeze-clamped, Langendorff-perfused whole hearts measured by SAMS peptide phophorylation assay in the presence of AMP (n = 5). (**g**) Histological appearances of hearts from Homo fl Cre+ and WT Cre+ mice aged 3 months stained with haematoxylin and eosin (H&E), sirius red and PAS (scale bar 50 μm). (**h-j**) Echocardiographic assessment of cardiac structure and function of Homo fl Cre+ mice versus WT Cre+ controls, with depiction of mean left ventricular (LV) posterior wall (PW) thickness (**h**), end-diastolic dimension (EDD) (**i**) and fractional shortening (FS) of LV mid-cavity (**j**). In **c-f, h-j,** the Student's *t*-test was performed. $^{**}P < 0.01$, $^{***}P < 0.001$. In **c-f, h-j,** data are shown as means ± s.e.m.

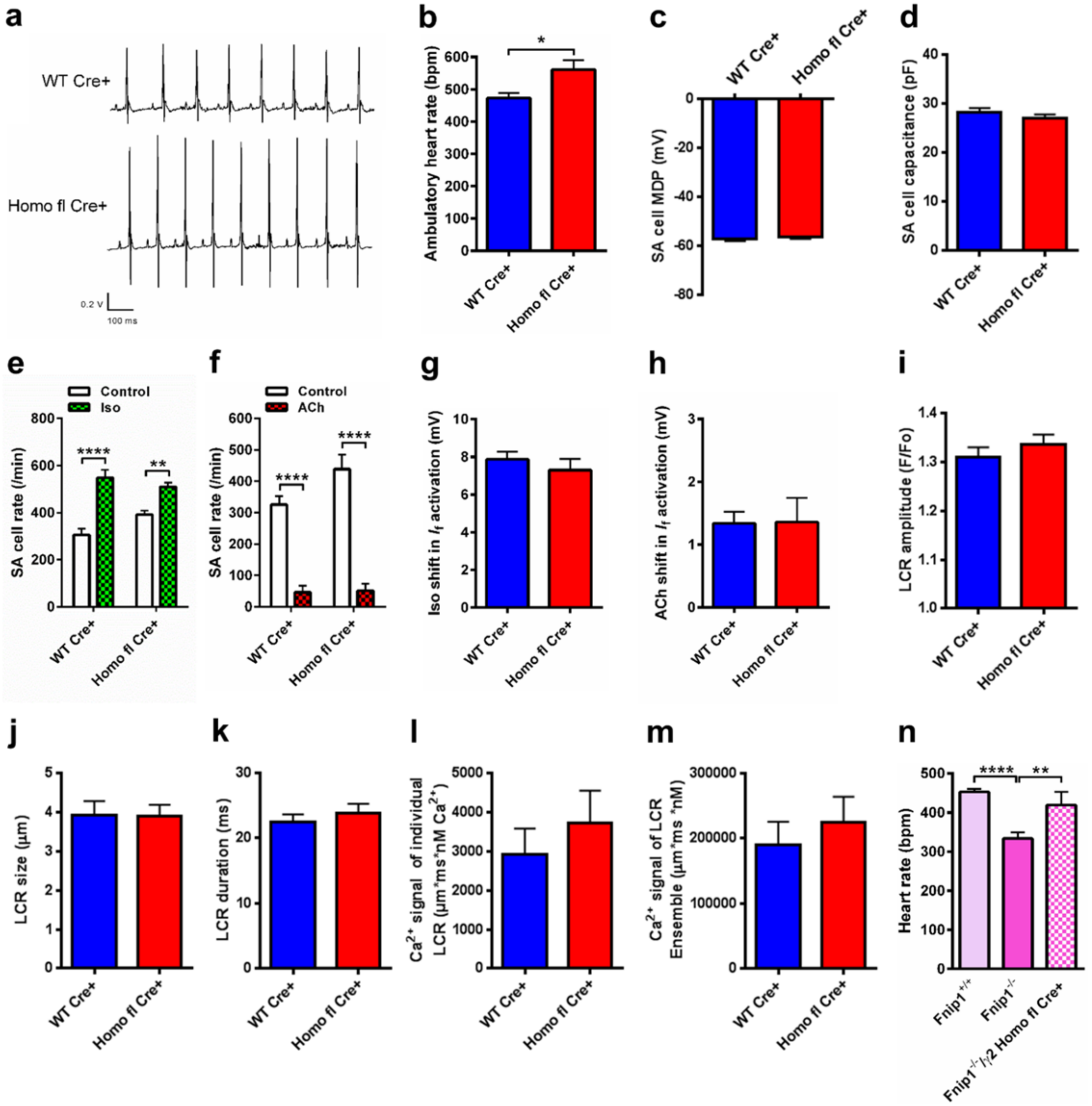
a
WT Cre+
Homo fl Cre+
0.2 V
100 ms
b
Ambulatory heart rate (bpm)
*
WT Cre+
Homo fl Cre+
c
SA cell MDP (mV)
WT Cre+
Homo fl Cre+
d
SA cell capacitance (pF)
WT Cre+
Homo fl Cre+
e
Control
Iso
SA cell rate (/min)
****
**
WT Cre+
Homo fl Cre+
f
Control
ACh
SA cell rate (/min)
****
****
WT Cre+
Homo fl Cre+
g
Iso shift in I_f activation (mV)
WT Cre+
Homo fl Cre+
h
ACh shift in I_f activation (mV)
WT Cre+
Homo fl Cre+
i
LCR amplitude (F/Fo)
WT Cre+
Homo fl Cre+
j
LCR size (µm)
WT Cre+
Homo fl Cre+
k
LCR duration (ms)
WT Cre+
Homo fl Cre+
l
Ca^{2+} signal of individual LCR (µm*ms*nM Ca^{2+})
WT Cre+
Homo fl Cre+
m
Ca^{2+} signal of LCR Ensemble (µm*ms *nM)
WT Cre+
Homo fl Cre+
n
Heart rate (bpm)
****
**
$Fnip1^{+/+}$
$Fnip1^{-/-}$
$Fnip1^{-/-}/_f2$ Homo fl Cre+

Supplementary Figure 9. Ambulatory heart rate and SA cell electrophysiology of γ2 AMPK knock-out mice. (**a, b**) Representative ambulatory telemetry-derived ECG recordings (**a**) and mean ambulatory HR (**b**) of Homo fl Cre+ and WT Cre+ mice (n = 6 per genotype). (**c, d**) SA cell maximum diastolic potential (MDP) (**c**) and capacitance (**d**) from Homo fl Cre+ and WT Cre+ mice. (**e, f**) Mean SA cell spontaneous rate under control conditions or in response to stimulation with 30 nM isoproterenol (Iso, **e**) or 30 nM acetylcholine (ACh, **f**) (n = 7-9). (**g, h**) Mean shifts of the I_f activation curve induced by 30 nM Iso (**g**) or 30 nM ACh (**h**). Iso and ACh shifts are depolarizing and hyperpolarizing, respectively (n = 5-8). (**i-k**) Mean spontaneous LCR amplitude (**i**), size (**j**) and duration (**k**) of isolated SA cells from Homo fl Cre+ and WT Cre+ mice (n = 28 cells/5 mice). (**l, m**) Ca^{2+} signal of individual LCRs (**l**) and LCR ensemble (**m**). (**n**) HR of WT FNIP (*Fnip1*$^{+/+}$), homozygous FNIP1 null (*Fnip1*$^{-/-}$) and compound homozygous FNIP1 null /Sox2cre γ2 AMPK knock-out mice (FNIP1$^{-/-}$/ γ2 Homo fl Cre+) mice under general anesthesia (n = 8-15). In **b-d, g-m,** the Student's t-test was performed; in **e, f,** two-way ANOVA was performed; in **n,** one-way ANOVA was performed. *$P < 0.05$, **$P < 0.01$, ****$P < 0.0001$. In **b-n,** data are shown as means ± s.e.m.

Fig. 1d

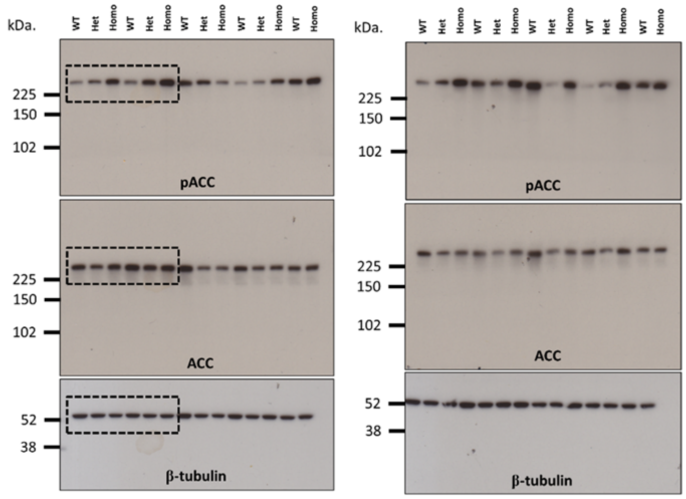

Fig. 1m

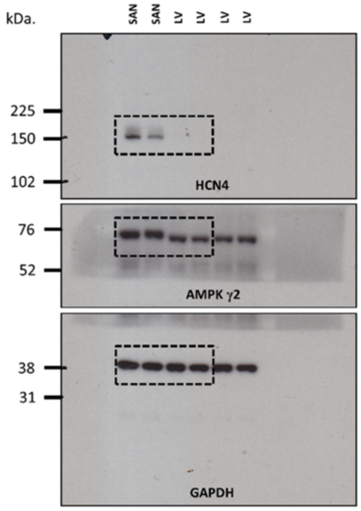

Fig. 1m

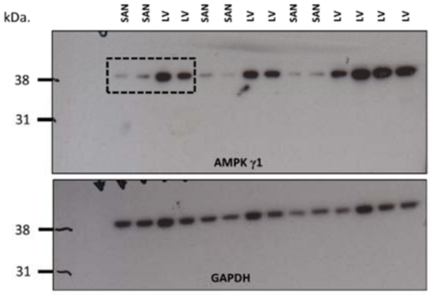

Fig. 1m

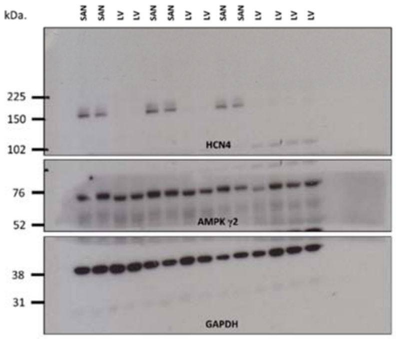

Fig. 1m

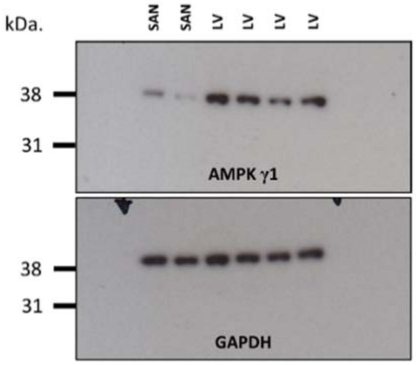

Fig. 2f

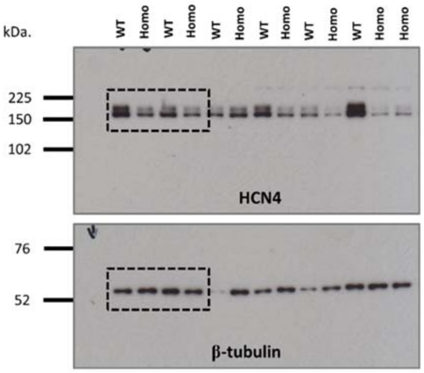

Fig. 7a

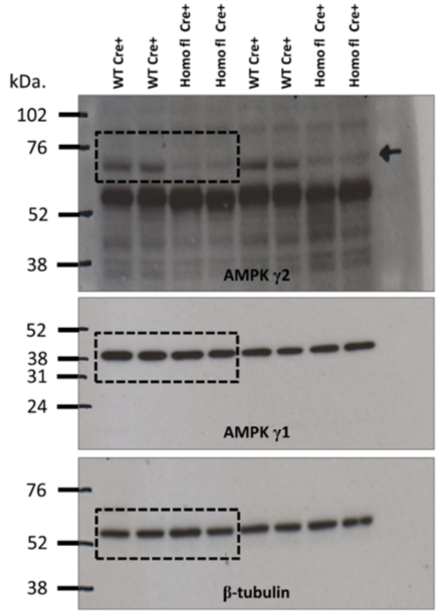

Supplementary Fig. 1b

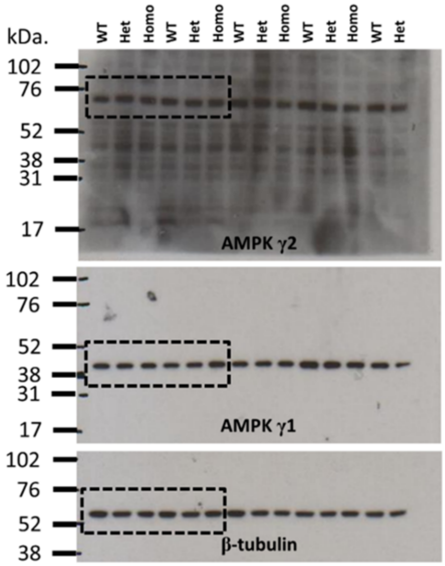

Supplementary Fig. 1k

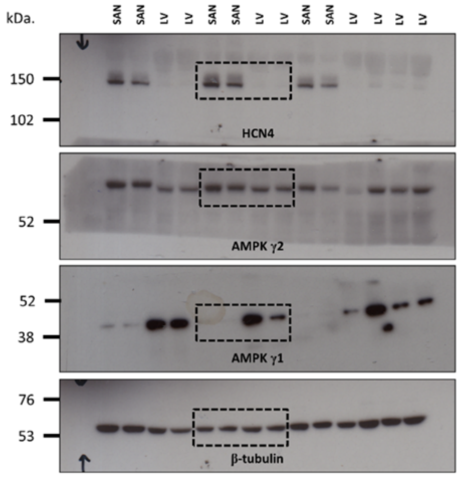

Supplementary Fig. 1n

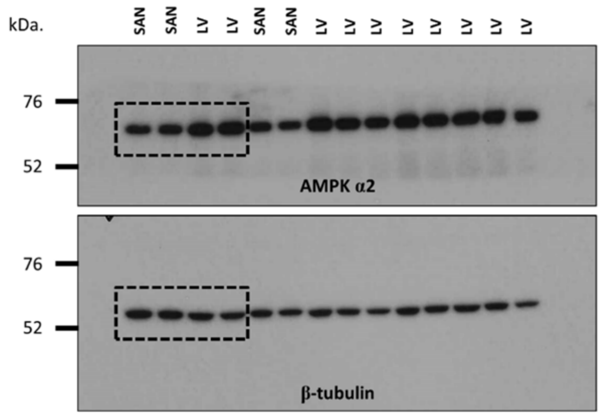

Supplementary Fig. 3j

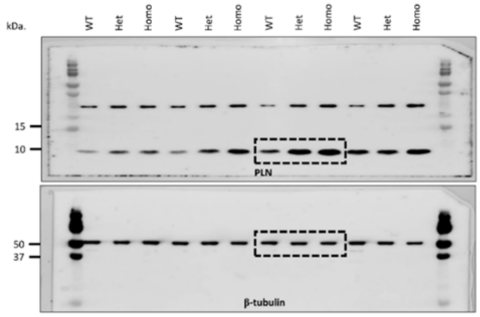

Supplementary Fig. 3k

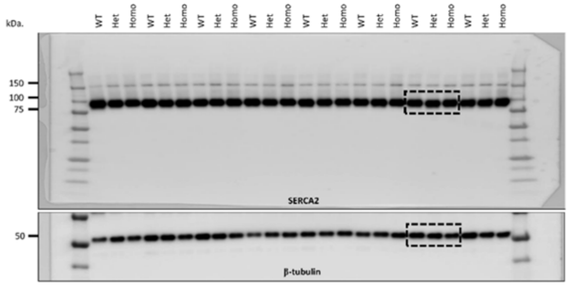

Supplementary Fig 3m.

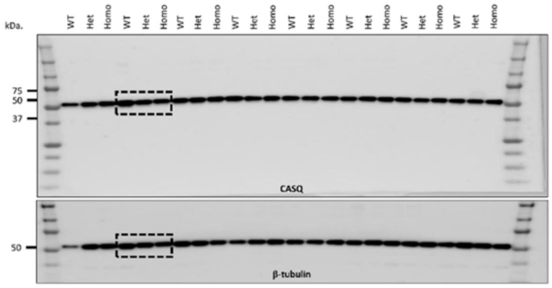

Supplementary Fig. 3n

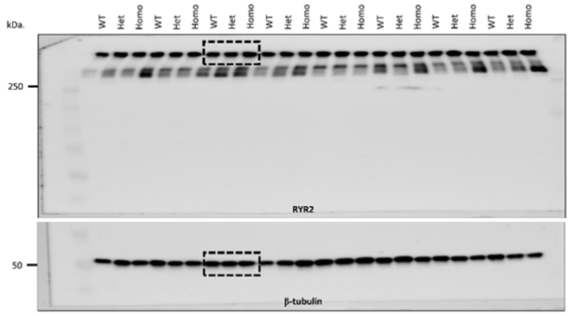

Supplementary Fig. 3o

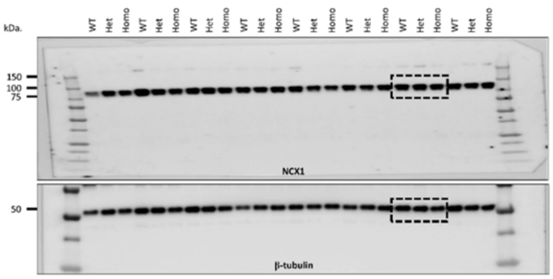

Supplementary Fig. 3p

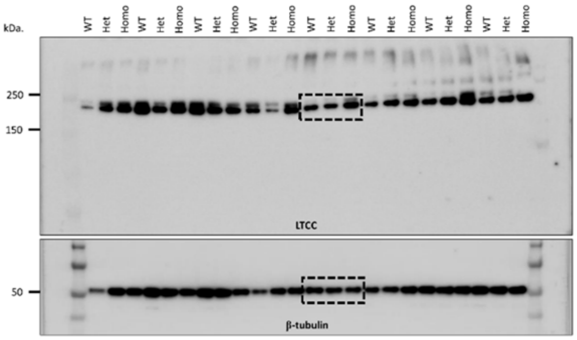

Supplementary Figure 10. Full length Western blots shown in main and Supplementary Figures. Cropped areas shown in the corresponding figure are indicated by dashed boxes.

Supplementary Table 1. Conduction parameters at invasive electrophysiology study of R299Q γ2 mice.

Intervals (in ms)	WT (n = 11)	Het (n = 7)	Homo (n = 13)
Baseline SCL	104.4 ± 2.7	112.3 ± 3.9	126.9 ± 3.6 *
SNRT	104.3 ± 11.2	110.1 ± 7.5	115.6 ± 6.3
AVW	67.6 ± 4.3	63.4 ± 3.7	67.3 ± 1.3
AV 2:1	53.1 ± 3.2	48.1 ± 2.8	52.5 ± 1.7
AVNERP	43.9 ± 4.6	39.2 ± 2.8	40.8 ± 2.4

SCL, Sinus cycle length; SNRT, sinus node recovery time; AVW, atrioventricular (AV) Wenckebach cycle length; AV 2:1, AV 2:1 cycle length; AVNERP, AV nodal effective refractory period. *$P < 0.05$ vs Het and $P < 0.001$ vs WT. Data are shown as mean ± s.e.m.

Supplementary Table 2. Top 50 genes downregulated in SA node of R299Q γ2 homozygote versus WT mice.

Gene	Gene Title	Score
Prkag2	protein kinase, AMP-activated, gamma 2 non-catalytic subunit	-0.594
Thumpd1	THUMP domain containing 1	-0.409
Ppp1r1b	dopamine and cAMP regulated phosphoprotein, DARPP-32	-0.315
Bdh1	3-hydroxybutyrate dehydrogenase, type 1	-0.300
Yipf7	Yip1 domain family, member 7	-0.266
Cilp2	cartilage intermediate layer protein 2	-0.230
Gcgr	glucagon receptor	-0.188
Wfdc2	WAP four-disulfide core domain 2	-0.182
Rab3b	RAB3B, member RAS oncogene family	-0.170
Abat	4-aminobutyrate aminotransferase	-0.170
Gsg1	germ cell associated 1	-0.170
Isl1	ISL1 transcription factor, LIM/homeodomain, (islet-1)	-0.166
Gja5	gap junction protein, alpha 5, 40kDa (connexin 40)	-0.163
Tmprss13	transmembrane protease, serine 13	-0.162
Fcho1	FCH domain only 1	-0.152
Hist1h2be	histone cluster 1, H2be	-0.146
Lrrc49	leucine rich repeat containing 49	-0.142
Ramp1	receptor (calcitonin) activity modifying protein 1	-0.141
Tmem35	transmembrane protein 35	-0.138
Tmem100	transmembrane protein 100	-0.137
Xrcc2	X-ray repair complementing defective repair in Chinese hamster cells 2	-0.132
Olfm1	olfactomedin 1	-0.130
Sfrp5	secreted frizzled-related protein 5	-0.126
Tef	thyrotrophic embryonic factor	-0.126
Fmod	fibromodulin	-0.125
Rpp25	ribonuclease P 25kDa subunit	-0.122
Dapk2	death-associated protein kinase 2	-0.119
Tmem63b	transmembrane protein 63B	-0.118

Rassf2	Ras association (RalGDS/AF-6) domain family 2	-0.117
Pi16	peptidase inhibitor 16	-0.116
Hs3st1	heparan sulfate (glucosamine) 3-O-sulfotransferase 1	-0.115
Cuedc1	CUE domain containing 1	-0.114
Unc13b	unc-13 homolog B (C. elegans)	-0.113
Arc	activity-regulated cytoskeleton-associated protein	-0.112
Gstm2	glutathione S-transferase M2 (muscle)	-0.112
Chrm2	cholinergic receptor, muscarinic 2	-0.112
Tbx3	T-box 3 (ulnar mammary syndrome)	-0.112
Mmp9	matrix metallopeptidase 9	-0.111
Junb	jun B proto-oncogene	-0.111
Gstt1	glutathione S-transferase theta 1	-0.110
Trappc5	trafficking protein particle complex 5	-0.110
Pdlim4	PDZ and LIM domain 4	-0.110
Ncam1	neural cell adhesion molecule 1	-0.110
Cxcl14	chemokine (C-X-C motif) ligand 14	-0.110
Rps15a	ribosomal protein S15a	-0.110
Mapk10	mitogen-activated protein kinase 10	-0.110
Gnao1	guanine nucleotide binding protein, alpha activating activity polypeptide O	-0.109
Gng2	guanine nucleotide binding protein (G protein), gamma 2	-0.108
Clec10a	C-type lectin domain family 10, member A	-0.108
Igfbpl1	insulin-like growth factor binding protein-like 1	-0.108

Supplementary Table 3. Top 50 genes upregulated in SA node of R299Q γ2 homozygote versus WT mice.

Gene	Gene Title	Score
Ptgds	prostaglandin D2 synthase 21kDa (brain)	0.6245
Abhd1	abhydrolase domain containing 1	0.4233
Myl2	myosin, light chain 2, regulatory, cardiac, slow	0.3654
Nppb	natriuretic peptide precursor B	0.3467
Myl3	myosin, light chain 3, alkali; ventricular, skeletal, slow	0.3325
Psca	prostate stem cell antigen	0.2587
Ankrd23	ankyrin repeat domain 23	0.2378
Igfals	insulin-like growth factor binding protein, acid labile subunit	0.2337
Cplx1	complexin 1	0.2246
Cxcl9	chemokine (C-X-C motif) ligand 9	0.2112
Aldob	aldolase B, fructose-bisphosphate	0.2025
Npy	neuropeptide Y	0.1891
Syt4	synaptotagmin IV	0.1745
Cartpt	CART prepropeptide	0.1722
Ivns1abp	influenza virus NS1A binding protein	0.1559
Rsad2	radical S-adenosyl methionine domain containing 2	0.1542
Lrrc10	leucine rich repeat containing 10	0.1498
Stmn2	stathmin-like 2	0.1419
Pfkfb2	6-phosphofructo-2-kinase/fructose-2,6-biphosphatase 2	0.1399
Ddit4	DNA-damage-inducible transcript 4	0.1374
Tyrp1	tyrosinase-related protein 1	0.1362
Fgf7	fibroblast growth factor 7 (keratinocyte growth factor)	0.1361
Apoc1	apolipoprotein C-I	0.1356
Phkb	phosphorylase kinase, beta	0.1346
Sox17	SRY (sex determining region Y)-box 17	0.1345
Pten	phosphatase and tensin homolog (mutated in multiple advanced cancers 1)	0.1334
Tmem106b	transmembrane protein 106B	0.1327
Asb10	ankyrin repeat and SOCS box-containing 10	0.1323

Gpr158	G protein-coupled receptor 158	0.1307
Sult4a1	sulfotransferase family 4A, member 1	0.1276
Arrdc4	arrestin domain containing 4	0.1269
Cidec	cell death-inducing DFFA-like effector c	0.1261
Vgll2	vestigial like 2 (Drosophila)	0.1240
Slc10a6	solute carrier family 10 (sodium/bile acid cotransporter family), member 6	0.1238
Rtn4	reticulon 4	0.1229
Ndfip2	Nedd4 family interacting protein 2	0.1223
Adamts8	ADAM metallopeptidase with thrombospondin type 1 motif, 8	0.1185
Rtn1	reticulon 1	0.1178
Pcdh7	BH-protocadherin (brain-heart)	0.1171
Psmd6	proteasome (prosome, macropain) 26S subunit, non-ATPase, 6	0.1168
Per2	period homolog 2 (Drosophila)	0.1160
Eif4e	eukaryotic translation initiation factor 4E	0.1150
Slc38a2	solute carrier family 38, member 2	0.1146
Pccb	propionyl Coenzyme A carboxylase, beta polypeptide	0.1142
Stat1	signal transducer and activator of transcription 1, 91kDa	0.1140
Jak1	Janus kinase 1 (a protein tyrosine kinase)	0.1139
Fkbp5	FK506 binding protein 5	0.1135
Ptprb	protein tyrosine phosphatase, receptor type, B	0.1127
Slc40a1	solute carrier family 40 (iron-regulated transporter), member 1	0.1116
Pparg	peroxisome proliferative activated receptor, gamma	0.1109

Supplementary Table 4. Network screening analysis evaluation of sample genes associated positively with the green co-expression module.

Gene	*P* value	*q* value	Z-score
Prkag2	0.011	0.016	4.47
Tbx3	0.002	0.010	6.85
Tbx18	0.012	0.017	4.24
Hcn2	0.002	0.010	7.23
Hcn4	0.005	0.011	5.58
Prkar1a	0.004	0.010	6.08
Prkar2b	0.004	0.010	5.99
Prkcdbp	0.007	0.012	5.17
Plcb3	0.003	0.010	6.73
Camk2	0.003	0.010	6.68
Camkk2	0.002	0.010	6.92
Camk4	0.002	0.010	6.82
Calml4	0.002	0.010	6.84
Calcrl	0.002	0.010	6.84
Calhm2	0.002	0.010	7.20
Prkci	0.009	0.014	-4.67
Camta1	0.026	0.026	-3.47
Prkab1	0.008	0.013	-4.83

Supplementary Table 5. Primer sequences.

Genotyping primers		
**Name**	**Forward 5’ – 3’**	**Reverse 5’ – 3’**
R299Q *Prkag2*	CACCTGAAGTTGCCGTGTGACCTCC	GAGGCATTTCCTCAAGGGAGGCTCC
Sox2cre	CTGGTGTAGCTGATGATCCG	CTCAGTGGTATTTGTGAGCC
Intact exon 7	TGTTTCTGGTTTCTTTCCAGGTCAA	TGCAAAAGCCAGAAAGTTCTTCAAC
Excised exon 7	GGTGTGCACAGCATCCAGCAACA	AGGCTGAGCTCACCGTTACTCGTG
**Allelic discrimination primers**		
**Name**	**Forward 5’ – 3’**	**Reverse 5’ – 3’**
Exon-spanning	CTTCTTTGCCTTGGTAGCCAAC	CATTCCTACAAAGCTCTGCTTTTTACTT
**SA node microarray validation primers**		
**Gene**	**Forward 5’ – 3’**	**Reverse 5’ – 3’**
Acta1	CCACAGCTGAACGTGAGATTGT	TGGCCATCTCATTCTCGAAGT
Hcn1	CACAGTGCCACAACGTGTCA	CTGCATCTGGGTCTGTATTTAGGA
Hcn4	CCACCCCTACAGTGACTTCAGAT	CCCACGGGTATGATGATCAGA
Isl1	TGACTCCTGTCTGTCCAAGAACTT	CCAGAAAGAGCGAAAACAGTCATT
Myl3	CCCAAGGGCGAGATGAAGAT	TCTGCCTGGGTAGGATTCTGA
Nppb	GGTCTGGCCGGACACTCA	AGCTTGTGCACTGGTGTCTTCA
Pln	GTAGTTGGCCATGCTGAGAGAA	TGTGGTAGTGAGCAATATAACAAATGC
Ppp1r1b	GTCTCTGTTTGCTTTGGCTGAA	CCCTACTCCCACCGGGTAAA
Ptgds	CAAAGCTGGAGGGTGTAGAGGTA	ACTCCTTTGCTCTGTCACTCTCAA
Tbx3	TGCCTATCAGAATGACAAGATAACTCA	TGTCTCGAAAACCCTTTGCAA
