# Supplementary material for: Mammalian γ2 AMPK regulates intrinsic heart rate
Source: Nat Commun. 2017 Nov 2;8:1258. doi: 10.1038/s41467-017-01342-5 (PMC5668267; doi:10.1038/s41467-017-01342-5)
Supplement: Supplementary file 2 — Description of Additional Supplementary Files [file 41467_2017_1342_MOESM2_ESM.pdf]

Supplementary Movie legends

Supplementary Movie 1. Effect of 0.5 mM AICAR on iSAB spontaneous beating rate.

The same iSAB is shown before and after drug administration, as well as after washout. Scale bar: 50 µm.

Supplementary Movie 2. Effect of 100 µM compound 991 on iSAB spontaneous beating rate.

The same iSAB is shown before and after drug administration, as well as after washout. Scale bar: 50 µm.
